# A Single-Chain Variable Fragment Antibody Alleviates Inflammation and Apoptosis of Neurons by Inhibiting Tau Aggregation

**DOI:** 10.3390/biom15060872

**Published:** 2025-06-15

**Authors:** Zongbao Wang, Jingye Lin, Peipei Chang, Mingzhu Sun, Sen Li

**Affiliations:** Gene Engineering and Biotechnology Beijing Key Laboratory, National Demonstration Center for Experimental Life Sciences & Biotechnology Education, College of Life Sciences, Beijing Normal University, Beijing 100875, China; 202231200029@mail.bnu.edu.cn (Z.W.);

**Keywords:** Alzheimer’s disease, extracellular Tau aggregates, single-chain variable fragment antibody, ROS

## Abstract

Tau pathology is one of the main pathological features of Alzheimer’s disease (AD). Intracellular Tau may be released to the extracellular space upon neuron degeneration, where it has the potential to be toxic to other neurons. The propagation of Tau pathology, mediated by extracellular Tau aggregates, may underlie the pathogenesis of AD. Antibody therapies targeting Tau proteins are, therefore, considered highly promising. In this study, the cytotoxicity of extracellular Tau aggregates on SH-SY5Y cells was examined. The effect of extracellular Tau aggregates on intracellular Tau aggregation was also studied using a FRET-based assay. The extracellular Tau aggregates were found to cause intracellular Tau aggregation after entering the cells; meanwhile, ROS (reactive oxygen species) induced by Tau aggregates facilitated this process. A single-chain variable fragment antibody (scFv T1) inhibits Tau aggregation both extracellularly and intracellularly. ScFv T1 also inhibited the accumulation of ROS and alleviated the inflammation and apoptosis induced by Tau aggregates. These findings could provide experimental support for the study of neurotoxicity and related mechanisms of extracellular Tau aggregates, in addition to providing insights into the development of novel therapeutic agents to treat AD.

## 1. Introduction

The abnormal aggregation of Tau proteins is an important pathogenic factor leading to Alzheimer’s disease (AD), and the pathological burden caused by Tau aggregates has been highly correlated with the severity of cognitive impairment [1,2]. It is a well-established fact that nerve cell toxicity can be caused by intracellular Tau aggregates, but extracellular Tau aggregates have also been studied as important risk factors for AD in recent years [3,4,5], with research indicating that extracellular Tau can spread in the brain as pathological seeds [6]. In Alzheimer’s disease, the accumulation of extracellular oligomeric Tau in synapses is an early event in pathogenesis, and Tau pathology may progress through the brain via trans-synaptic spread [7]. Extracellular Tau is more toxic to neurons and was found to spread between nerve cells in a mouse model of Tau disease [8,9]. Additionally, extracellular Tau aggregates were found to lead to synaptic dysfunction, synaptic loss, and inflammation processes [10]. Moreover, extracellular Tau aggregates not only disrupt the astrocytic mitochondrial system and alter mitochondrial morphology but also stimulate microglia to phagocytose live neurons [11,12]. Thus, the neurotoxicity and related mechanisms of extracellular Tau aggregates are worth further investigation.

Due to the close relationship between extracellular Tau and AD, Tau-binding agents that can access extracellular Tau, such as anti-Tau antibodies, might be valuable tools for preventing the uptake of extracellular Tau into neurons and decreasing the spread of Tau pathology. Recently, passive immunization with different anti-Tau antibodies was shown to have beneficial effects against Tau pathology [13,14,15]. In line with this, we previously screened a single-chain variable fragment antibody (scFv T1) that could inhibit the aggregation of Tau in vitro and ameliorate its cytotoxicity, while ensuring that the presence of scFv T1 did not inhibit Tau-induced tubulin assembly [16]. As SH-SY5Y cells possess a range of neurobiological properties and have been extensively utilized in experimental studies pertaining to diseases of the nervous system [17], we examined the cytotoxicity of extracellular Tau aggregates on SH-SY5Y cells in this study. It was found that extracellular Tau aggregates caused intracellular Tau aggregation after entering the cells, and ROS promoted the aggregation process. Such effects could result in the inflammation and apoptosis of SH-SY5Y cells. Furthermore, the effect of scFv T1 was studied, and it was found to inhibit the aggregation of Tau in cells and alleviate the inflammation and apoptosis induced by Tau aggregates.

## 2. Materials and Methods

### 2.1. Materials

ECFP-C1 plasmid was obtained from Clontech, Mountain View, CA, USA (Clontech Cat# PT3259-5). EYFP-C1 plasmid was obtained from Clontech (Clontech Cat# PT3176-5), Thioflavin T was obtained from Sigma, Oakville, ON, Canada (Sigma Cat# T3516), and heparin was obtained from Sangon Biotech, Shanghai, China (Sangon Biotech Cat# A603251-0001). Both the SH-SY5Y cells and HEK-293T cells used in this study were obtained from Pricella, Wuhan, China (Pricella Cat# CL-0208 and Pricella Cat# CL-0005, respectively), and the FITC conjugation kit used in our research was obtained from Sangon Biotech (Sangon Biotech Cat# D601049-0001).

### 2.2. Expression and Purification of Tau Protein

The DNA encoding human Tau 40 (residues 1–441) was subcloned into a PET-31b (+) vector, and the recombinant plasmid was transformed into the *E. coli* BL21(DE3) strain (TransGen Biotech, Beijing, China, TransGen Biotech Cat# CD601-02). The expression of recombinant human full-length Tau was induced with the use of 0.4 mM IPTG for 16 h at 37 °C, after which the cells were lysed via sonication. After centrifugation at 12,000 rpm for 20 min at 4 °C, the supernatant was boiled for 10 min and centrifuged to remove all insoluble proteins. After centrifugation at 12,000 rpm for 20 min at 4 °C, the supernatant was collected and passed through a 0.45 µm pore size filter to remove the insoluble particles. The remaining soluble protein was then purified using a Ni Sepharose 6 Fast Flow column (Cytiva, Marlborough, MA, USA, Cytiva Cat# 17531801).

### 2.3. Expression and Purification of scFv T1

The scFv T1 DNA was subcloned into a pET-28a (+) vector, and the recombinant plasmid was transformed into the *E. coli* BL21(DE3) strain. The expression of scFv T1 was induced with the use of 1 mM IPTG for 16 h at 16 °C, after which the cells were lysed via sonication. The supernatant was collected at 12,000 rpm for 20 min at 4 °C and filtered through a 0.45 µm pore size filter. The filtrate was then purified using the Ni Sepharose 6 Fast Flow column (Cytiva Cat# 17531801).

### 2.4. Tau Aggregates Preparation

Investigating Tau aggregation in vitro has required the addition of polyanions or other co-factors, with heparin being the most commonly used [18]. Thus, heparin control samples were incubated without agitation in a buffer (10 µM heparin, 17.7 mM NaCl, 100 µM DTT, and 10 mM HEPES, pH 7.4) at 37 °C for 48 h. Tau protein (10 μM) samples were also incubated without agitation in the aggregation buffer (10 µM heparin, 17.7 mM NaCl, 100 µM DTT, and 10 mM HEPES, pH 7.4) at 37 °C for 48 h to obtain aggregates. To study the effect of scFv T1 on Tau aggregation, the Tau proteins (10 µM) were incubated with scFv T1 (10 µM, 15 µM, and 20 µM) in the same buffer for 48 h. To study the effect of scFv T1 on neurotoxicity caused by extracellular Tau aggregates, the Tau proteins (10 µM) were incubated with scFv T1 (15 µM) in the same buffer for 48 h.

### 2.5. ThT Assay

The Tau proteins (10 μM) and ThT (20 μM) were mixed at 37 °C in a buffer (10 µM heparin, 17.7 mM NaCl, 100 µM DTT, and 10 mM HEPES, pH 7.4). ScFv T1 (10 μM), Tau proteins (10 μM), and ThT (20 μM) were mixed at 37 °C in the buffer (10 µM heparin, 17.7 mM NaCl, 100 µM DTT, and 10 mM HEPES, pH 7.4), and fluorescence was subsequently measured (λex 440 nm; λem 520 nm) every hour as described.

### 2.6. LDH Cytotoxicity Assay

The SH-SY5Y cell line was obtained from Pricella (CL-0208). SH-SY5Y cells were cultured in DMEM/F12 supplemented with 10% FBS. All of the cells were divided into several groups. Cells in the normal control group, heparin treatment control group, Tau monomer treatment group, and Tau aggregate treatment group were treated with PBS, heparin (5 µM), Tau monomers (5 µM), or Tau aggregates (2.5–7.5 µM), respectively, for 24 h. By contrast, cells in the positive control group were treated with an LDH release agent for 1 h. Cytotoxicity was detected with an LDH assay kit (Beyotime, Shanghai, China, Beyotime Cat# C0016). LDH detection working solution was added and incubated for 30 min at 25 °C. The absorbance of the samples was then detected at 490 nm using a POLARstar Omega software V 5.10 R2 multimode microplate reader (BMG Labtech, Ortenberg, Germany, DMI3000B).

### 2.7. Live Cell Staining

The Tau proteins were conjugated with FITC to obtain FITC–Tau using an FITC conjugation kit (Sangon Biotech, D601049-0001). Then FITC–Tau (10 µM) was incubated without agitation in an aggregation buffer (10 µM heparin, 17.7 mM NaCl, 100 µM DTT, and 10 mM HEPES, pH 7.4) at 37 °C for 48 h to obtain FITC–Tau aggregates. FITC–Tau (10 µM) was incubated with scFv T1 (15 µM) in the same buffer for 48 h to obtain FITC–Tau–scFv T1 mixtures (Tau: 10 µM; scFv T1: 15 µM). SH-SY5Y cells were cultured in small dishes and treated with FITC–Tau monomers (5 µM), FITC–Tau aggregates (5 µM), or FITC–Tau–scFv T1 mixtures (FITC–Tau: 5 µM; scFv T1: 7.5 µM) for 24 h. The microtubule skeleton was stained with a microtubule far-infrared fluorescence staining kit (Beyotime Cat# C2215S). Nuclei were stained with Hoechst 33,342 live cell staining solution (Beyotime Cat# C1028). Lastly, the cells were placed in a serum-free medium and observed via laser confocal microscopy (ZEISS, Oberkochen, Germany, LSM880).

### 2.8. FRET-Based Assay

The HEK-293T cell line was obtained from Pricella (CL-0005). HEK-293T cells were cultured in DMEM supplemented with 10% FBS. The presence of intracellular Tau aggregates was then assessed via fluorescence resonance energy transfer (FRET). The DNA encoding Tau repeat domain (RD) with the disease-associated P301S mutation was reprogrammed into plasmids expressing ECFP (Enhanced Cyan Fluorescent Protein) or EYFP (Enhanced Yellow Fluorescent Protein). HEK-293T cells transfected with Tau RD/P301S-ECFP and Tau RD/P301S-EYFP recombinant plasmids using jetPRIME transfection reagent (Polyplus, Graffenstaden, France, Polyplus Cat# 101000046) were randomly divided into several groups, and a FRET assay was performed before treatment. Each group was treated with heparin (5 μM), NAC (500 μM), rotenone (5 μM), Tau aggregates (5 μM), Tau aggregates–NAC mixtures (Tau aggregates: 5 µM; NAC: 500 µM), and three Tau–scFv T1 co-incubation mixtures (Tau:scFv T1 = 5 μM:5 μM, 5 μM:7.5 μM and 5 μM:10 μM) separately for 24 h, after which the FRET assay was performed again. In the FRET assay, the fluorescence intensity of ECFP (excited with a 458 nm laser, with fluorescence captured via a 458–520 nm filter) and that of EYFP (fluorescence was captured with a 520–620 nm filter) was detected via laser confocal microscopy (ZEISS LSM880). FRET intensity was calculated as the ratio of the EYFP intensity to the ECFP intensity. The FRET intensity represents the strength of Tau aggregation. Each experiment was performed using ~10,000 cells per replicate, and each sample was analyzed in triplicate. Twenty cells were randomly selected to calculate the FRET intensity for each replicate. The FRET intensities of each group were normalized according to the intensities detected before they were processed. Data analyses were performed with Image J software V 2.9.0 and the statistical analysis software GraphPad Prism 8.

In the scFv T1 intracellular treatment groups, the Tau RD/P301S-ECFP, Tau RD/P301S-EYFP, and scFv-T1 plasmids were concurrently transfected into HEK-293T cells at different DNA ratios (Tau RD/P301S-ECFP:Tau RD/P301S-EYFP: scFv T1 = 0.3 μg:0.3 μg:0.6 μg, 0.3 μg:0.3 μg:0.9 μg, and 0.3 μg:0.3 μg:1.2 μg). All of the cells were treated with Tau aggregates for 24 h, after which the FRET assay was performed.

### 2.9. ROS Assay

The SH-SY5Y cells were cultured in DMEM/F12 supplemented with 10% FBS. The cells were divided into several groups and separately treated with PBS, heparin (5 µM), Tau aggregates (5 µM), and Tau–scFv T1 mixtures (Tau: 5 µM, scFv T1: 7.5 µM) separately for 24 h. The cells in the ROS-positive group were treated with Rosup (Beyotime Cat# S0033S) for 30 min, and ROS levels were detected with a ROS detection kit (Beyotime Cat# S0033S). The cells were first incubated with the probe DCFH-DA for 20 min at 37 °C. After the cells were washed three times with PBS, the fluorescence of the probe was detected via inverted fluorescence microscopy (ZEISS Observer Z1). The experiment was performed in triplicate, and each experiment was performed using 10,000 cells per replicate. Three fields of view were randomly selected for statistics. The total fluorescence intensity of the cells in each field of view was measured using Image J software V 2.9.0. Statistical analyses were then performed with the use of GraphPad Prism 8 software.

### 2.10. qPCR Analysis

Total RNA was extracted from the SH-SY5Y cells using a TRIzol kit (TransGen Biotech Cat# ET111-01). Reverse transcription was performed using 2 µg of total RNA via FastKing gDNA dispelling RT SuperMix (TIANGEN, Beijing, China, TIANGEN Cat# KR118-02). Real-time qPCR was performed using PerfectStart Green qPCR SuperMix (TransGen Biotech Cat# AQ602) and an ABI Quant Studio 6 Flex Real-Time PCR System (Thermo Fisher, Waltham, MA, USA, QuantStudio™ 6). After preincubation at 50 °C for 2 min and 95 °C for 30 s, the PCR reaction was performed as 40 cycles of 95 °C for 5 sec and 60 °C for 30 s. The experiment was performed in triplicate. Subsequently, the data were analyzed using the statistical analysis software GraphPad Prism 8.

The primers used were as follows: TNF-α forward primer: 5′-CCCAGGCAGTCAGATCATCTTCT-3′; TNF-α reverse primer: 5′-ATGAGGTACAGGCCCTCTGAT-3′; IL-6 forward primer: 5′-ACTCACCTCTTAGAACGAATTG-3′; IL-6 reverse primer: 5′-CCATCTTTGGAAGGTTCAGGTTG-3′; IL-1β forward primer: 5′-ATGATGGCTTATTACAGTGGCAA-3′; IL-1β reverse primer: 5′-GTCGGAGATTCGTAGCTGGA-3′; β-actin forward primer: 5′-GATGCAGAAGGAGATCACTGC-3′; β-actin reverse primer: 5′-ATACTCCTGCTTGCTGATCCA-3′.

### 2.11. Western Blot Analysis

The SH-SY5Y cells were lysed with RIPA buffer, and cell-soluble samples were boiled with a 6× protein loading buffer for 5 min before being subjected to SDS–PAGE.

HEK-293T cells transfected with Tau RD/P301S-ECFP and Tau RD/P301S-EYFP recombinant plasmids were randomly divided into several groups, and they were then treated with PBS, heparin, Tau aggregates, or Tau monomers for 24 h. The HEK-293T cells were then lysed with RIPA buffer. The cell lysates were centrifuged at 12,000× *g* for half an hour at 4 °C to remove the cell debris. Half of the supernatant was incubated with 1% sarkosyl for half an hour at 25 °C. The mixture was then ultracentrifuged at 160,000 g for half an hour, and the pellets were washed twice with 1 × PBS (pH 7.4). The sarkosyl-insoluble pellets were sonicated and boiled in SDS–PAGE loading buffer for 10 min and subjected to SDS–PAGE. The other half of the supernatant, which served as the total protein sample, was also boiled in the SDS–PAGE loading buffer for 10 min and subjected to SDS–PAGE.

Proteins in the gel were transferred to PVDF membranes (Millipore, Bayswater, Australia, Millipore Cat# IPVH00010), which were blocked with QuickBlock™ Blocking Buffer (Beyotime Cat# P0252) followed by overnight incubation with primary antibodies (anti-β-actin, 1:1000, Abcam Cat# 8226; anti-GAPDH, 1:1000, Proteintech Cat# 10494-1-AP; anti-Flag (DYKDDDDK), 1:1000, Proteintech Cat# 20543-1-AP; anti-Bax, 1:1000, AbMART, Shanghai, China, Abmart Cat# T40051F; anti-Bcl-2, 1:1000, Abmart Cat# T40056F; anti-Caspase-3, 1:1000, Abmart Cat# T40044F; anti-NF-κB p65, 1:1000, Abmart Cat# T55034F; anti-p-NF-κB p65 (Ser529), 1:500, Abmart Cat# TP56371F and anti-Tau 4R antibody, 1:1000, Abcam Cat# AB218314) diluted in QuickBlock™ Primary Antibody Dilution Buffer (Beyotime Cat# P0256). The membranes were subsequently washed three times with TBST for 10 min each time and then incubated with HRP-conjugated secondary antibodies (goat anti-mouse HRP-conjugated, 1:7500, BBI Cat# D110087-0100; goat anti-rabbit HRP conjugated, 1:7500, proteintech Cat# SA00001-2) for 1 h at RT and washed three times with TBST for 10 min each time. Lastly, the blot bands were visualized with the use of a chemiluminescent HRP-substrate (Millipore Cat# P90719) and analyzed using the ChemiDoc^TM^ XRS+ system with Image LabTM Software V 3.0 (Bio-Rad, Hercules, CA, USA, ChemiDoc MP). Western blot original images can be found in Supplementary Materials. The fold change in the protein levels was calculated using the following method: The protein level in each group (target protein/β-actin or GAPDH) was normalized according to that of the NC group. The amount of insoluble Tau aggregates in the HEK-293T cells transiently expressing Tau RD/P301S-ECFP and Tau RD/P301S-EYFP was determined as a ratio of the density of insoluble Tau aggregate bands over that of the total Tau bands in cell lysates; then, it was normalized according to that of the NC group. Statistical analyses were subsequently performed with the use of GraphPad Prism 8 software.

### 2.12. Statistical Analysis

All the statistical analyses were performed with the use of GraphPad Prism 8 software. The statistical analysis was performed using Student’s *t*-test or one-way ANOVA. The data are presented as the mean ± SD, with a *p* value < 0.05 considered significant. *p* values are listed as follows: * *p* < 0.05, ** *p* < 0.01, *** *p* < 0.001, ^#^ *p* < 0.05, ^##^ *p* < 0.01, ^###^ *p* < 0.001, ns, not significant.

## 3. Results

### 3.1. Extracellular Tau Aggregates Cause Inflammation and Apoptosis

**Figure 1:**
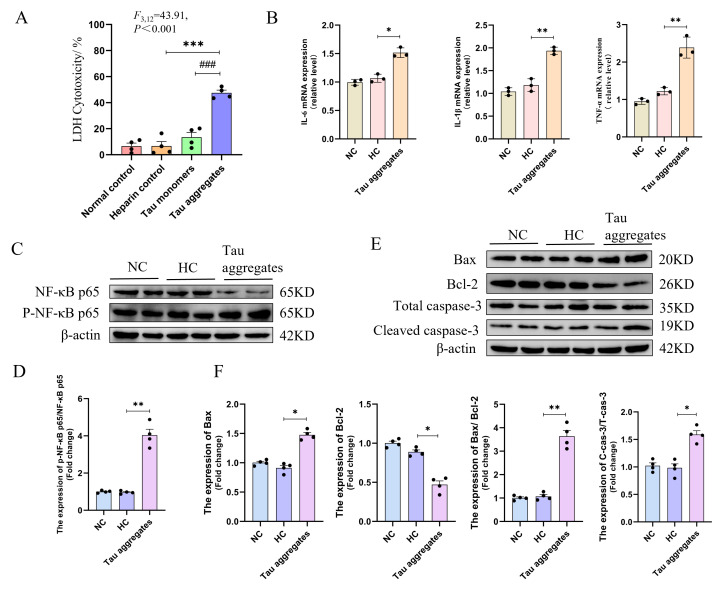
Extracellular Tau aggregates cause inflammation and apoptosis of SH-SY5Y cells. (**A**) The cytotoxicity of different samples to SH-SY5Y cells detected by the LDH method. This experiment was repeated four times. (**B**) The mRNA levels of IL-6, IL-1β, and TNF-α detected by qPCR. The experiment was performed in triplicate. (**C**) The protein levels of NF-kB p65 and p-NF-kB p65 detected by Western blot. The grouping of blots cropped from different parts of the same gel. The experiment was repeated four times. (**D**) Bar chart of the relative protein expression levels in (**C**). (**E**) The expression levels of Bax, Bcl-2, caspase-3, and cleaved caspase-3 detected by Western blot. The grouping of blots cropped from different parts of the same gel. The experiment was repeated four times. (**F**) Bar chart of the relative protein expression levels in (**E**). Data represent means ± SD and were analyzed by one-way ANOVA (**A**) or Student’s *t*-test (**B**,**D**,**F**). * *p* < 0.05, ** *p* < 0.01, *** *p* < 0.001, ^###^ *p* < 0.001.

Extracellular Tau aggregates could act as pathological seeds to cause damage to neurons. To investigate the effects of extracellular Tau aggregates on neurons, Tau monomers were induced to aggregate by heparin in vitro, and SH-SY5Y cells were then treated with the induced Tau aggregates for 24 h. The result of the ThT assay verified the formation of Tau aggregates (Figure S1). The effect of extracellular Tau aggregates on the viability of SH-SY5Y cells was measured using the LDH method. The results show that there is a significant cytotoxicity of 5 µM extracellular Tau aggregates on SH-SY5Y cells (Figure 1A and Figure S2A). Inflammation is the most important feature of cytotoxicity and has been found in the brains of patients with AD [19]. To explore the pro-inflammatory effect of Tau aggregates on nerve cells, the mRNA levels of the inflammatory cytokines IL-6, IL-1β, and TNF-α in SH-SY5Y cells were measured after Tau aggregate treatment. The results show that, compared with the PBS treatment normal control (NC) group and the heparin treatment control (HC) group, the mRNA levels of IL-6, IL-1β, and TNF-α in SH-SY5Y cells significantly increased in the Tau aggregate treatment group (Figure 1B). Additionally, as activation of the NF-κB pathway is associated with inflammation [20], the NF-κB pathway was examined, with results showing that the protein level of p-NF-κB p65 was increased in cells of the Tau aggregate treatment group compared to the heparin treatment group (Figure 1C,D). These results suggest that extracellular Tau aggregates could induce neuron inflammation. Moreover, inflammation in cells is often accompanied by apoptosis. Therefore, apoptosis-related factors were also examined. The results showed that the expression levels of Bax and cleaved caspase-3 increased in the Tau aggregate treatment group, whereas the expression level of bcl-2 decreased (Figure 1E,F) compared to that of the control groups. These results indicate that extracellular Tau aggregates induce the apoptosis of SH-SY5Y cells.

### 3.2. Extracellular Tau Aggregates Enter SH-SY5Y Cells and Induce Tau Aggregation in Cells

**Figure 2:**
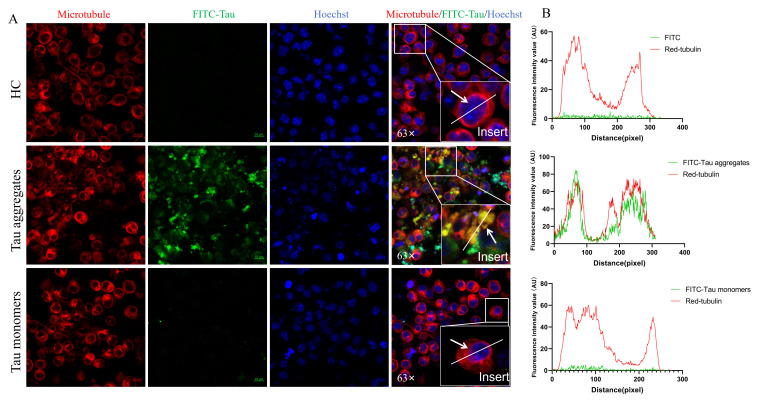
Extracellular Tau aggregates enter SH-SY5Y cells. (**A**) Representative confocal images showing colocalization of SH-SY5Y tubulin and FITC–Tau. The experiment was performed in triplicate. Red fluorescence: microtubule stained with far-infrared fluorescence staining kit; green fluorescence: FITC–Tau; blue fluorescence: nuclei stained with Hoechst 33342. Scale bar, 10 μm. (**B**) Intensity wave showing the colocalization of FITC–Tau and Red-tubulin at the white arrow.

Extracellular Tau aggregates are toxic to nerve cells, and the transmission of toxicity within the brains of patients with AD has been proposed to be caused by the spread of Tau [21]. Holmes et al. reported that extracellular Tau aggregates can enter cells [22], so we consider that extracellular Tau aggregates induce the aggregation of intracellular Tau monomers after entering cells. To investigate whether extracellular Tau proteins enter the cell for propagation in our experiment, Tau proteins were coupled to fluorescein isothiocyanate (FITC) and induced to form FITC–Tau aggregates via heparin. After the SH-SY5Y cells were treated with FITC–Tau aggregates or FITC–Tau monomers for 24 h, microtubules and nuclei were stained, after which the fluorescence signals of FITC–Tau, microtubules, and nuclei were detected. The results demonstrated that some FITC–Tau aggregates were colocalized to microtubules; however, the FITC–Tau monomers were not colocalized to the microtubules (Figure 2A,B and Figure S3). Therefore, these results indicate that parts of Tau aggregates enter SH-SY5Y cells.

**Figure 3:**
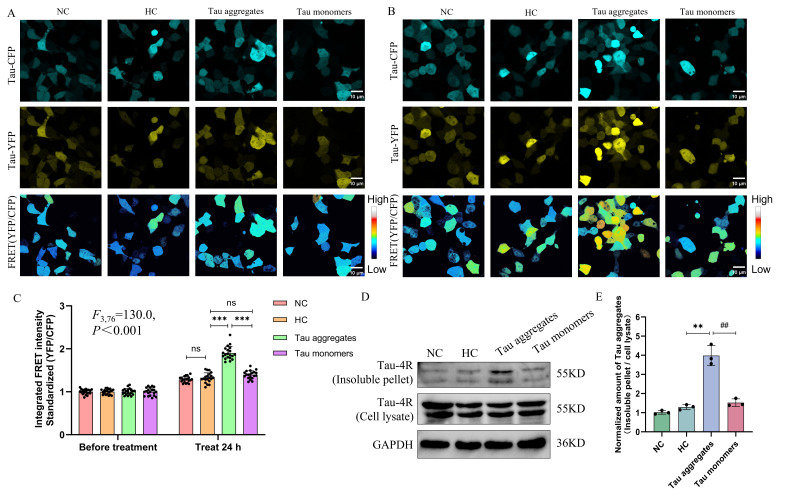
Extracellular Tau aggregates induce the aggregation of intracellular Tau protein. (**A**) Representative confocal micrograph of Tau-ECFP, Tau-EYFP, and FRET intensity in HEK-293T cells before treatment. Tau-ECFP: the fluorescence was excited with a 458 nm laser and captured with a 458–520 nm filter. Tau-EYFP: the fluorescence was captured with a 520–620 nm filter. FRET (EYFP/ECFP): FRET intensity was calculated as the ratio of the EYFP intensity to the ECFP intensity. Scale bar, 10 μm. (**B**) Representative confocal micrograph of Tau-ECFP, Tau-EYFP, and FRET intensity in HEK-293T cells after treatment. Scale bar, 10 μm. (**C**) FRET intensity quantification bar chart for (**A**,**B**). (**D**) The Tau 4R protein levels of sarkosyl-insoluble pellets and cell lysates in HEK-293T cells detected by Western blot. The experiment was repeated three times. The grouping of blots cropped from different gels of the same samples. (**E**) Bar chart of the relative protein expression levels in (**D**). Data represent means ± SD and were analyzed by one-way ANOVA (**C**) or Student’s *t*-test (**E**). ** *p* < 0.01, *** *p* < 0.001, ^##^ *p* < 0.01, ns, not significant.

Next, we investigated whether the Tau aggregates could induce the aggregation of intracellular Tau monomers after entering the cells. Fluorescence resonance energy transfer (FRET) is an effective powerful tool for studying macromolecular interactions such as protein–protein interactions. When a donor molecule (Enhanced Cyan Fluorescent Protein, ECFP) is close to or binds to an acceptor molecule (Enhanced Yellow Fluorescent Protein, EYFP), the stimulated donor molecule (ECFP) transfers the stimulated static energy to the acceptor molecule (EYFP) [23]. FRET has been widely used in the study of protein–protein interactions including protein aggregation in living cells [24]. We used a FRET-based assay to test the aggregation of intracellular Tau proteins. The repetitive fragments of Tau proteins (especially the repetitive regions in the microtubule-binding domain) are prone to aggregation under pathological conditions. In addition, Tau with the P301S mutation associated with FTDP-17 is prone to aggregation [25,26]. Thus, the Tau repeat domain (RD) with the P301S mutation was used for fusion to either ECFP or EYFP in order to study the aggregation of Tau in cells. The fluorescence intensities of ECFP and EYFP were detected after Tau RD/P301S-ECFP and Tau RD/P301S-EYFP were both expressed in HEK-293T cells, and the FRET intensity (the ratio of EYFP fluorescence intensity to ECFP fluorescence intensity) represents the degree of intracellular Tau RD/P301S aggregation. The results of the LDH assay showed the cytotoxicity of 5 μM Tau aggregates to HEK-293T cells (Figure S2B). The FRET assay results showed that there were no significant differences in FRET intensity between the NC group, HC group, Tau aggregate treatment group, and Tau monomer treatment group before the HEK-293T cells were treated with PBS, heparin, or Tau aggregates (Figure 3A,C). After the cells were treated for 24 h, no significant change in FRET intensity was found for the cells in the NC group or the HC group compared with that of the cells before treatment. However, the FRET intensity of the cells in the Tau aggregate treatment group was significantly higher than that of the cells before treatment (Figure 3B,C). No significant change in FRET intensity was found for the cells in the Tau monomer treatment group compared to that of the HC group after treatment (Figure 3B,C).

To gain a quantitative understanding of the effect of extracellular Tau aggregates on intracellular Tau aggregation, we performed Western blot analysis to detect insoluble Tau aggregates in the sarkosyl-insoluble ultracentrifugation pellets using anti-Tau 4R antibody. We found that the level of insoluble Tau aggregates in the HEK-293T of the Tau aggregate treatment group was significantly higher than that of the NC group, the HC group, and the Tau monomer treatment group (Figure 3D,E). This is consistent with the results of the FRET experiment. These results indicate that the extracellular Tau aggregates induce the aggregation of intracellular Tau proteins.

### 3.3. ROS (Reactive Oxygen Species) Are Important Risk Factors in the Process of Tau Aggregation in Cells

**Figure 4:**
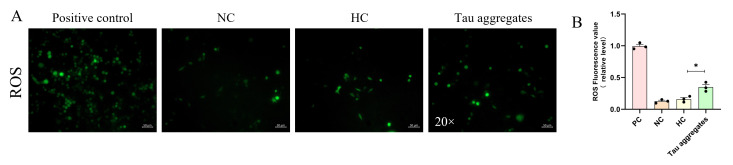
Extracellular Tau aggregates cause ROS accumulation. (**A**) Representative fluorescence micrograph showing ROS levels. Scale bar, 50 μm. (**B**) Bar chart of the relative ROS levels in (**A**). The experiment was performed in triplicate. Data represent means ± SD and were analyzed by Student’s *t*-test. * *p* < 0.05.

Oxidative stress and the accumulation of ROS are important pathological indicators in patients with AD and are also significant causes of inflammation and apoptosis [27]. Thus, to investigate the relationship between extracellular Tau aggregates and ROS, SH-SY5Y cells were treated with Tau aggregates for 24 h, after which the ROS levels in the cells were measured. The results showed that the ROS levels significantly increased after 24 h of treatment with the Tau aggregates (Figure 4A,B). This finding suggests that extracellular Tau aggregates lead to the accumulation of ROS.

**Figure 5:**
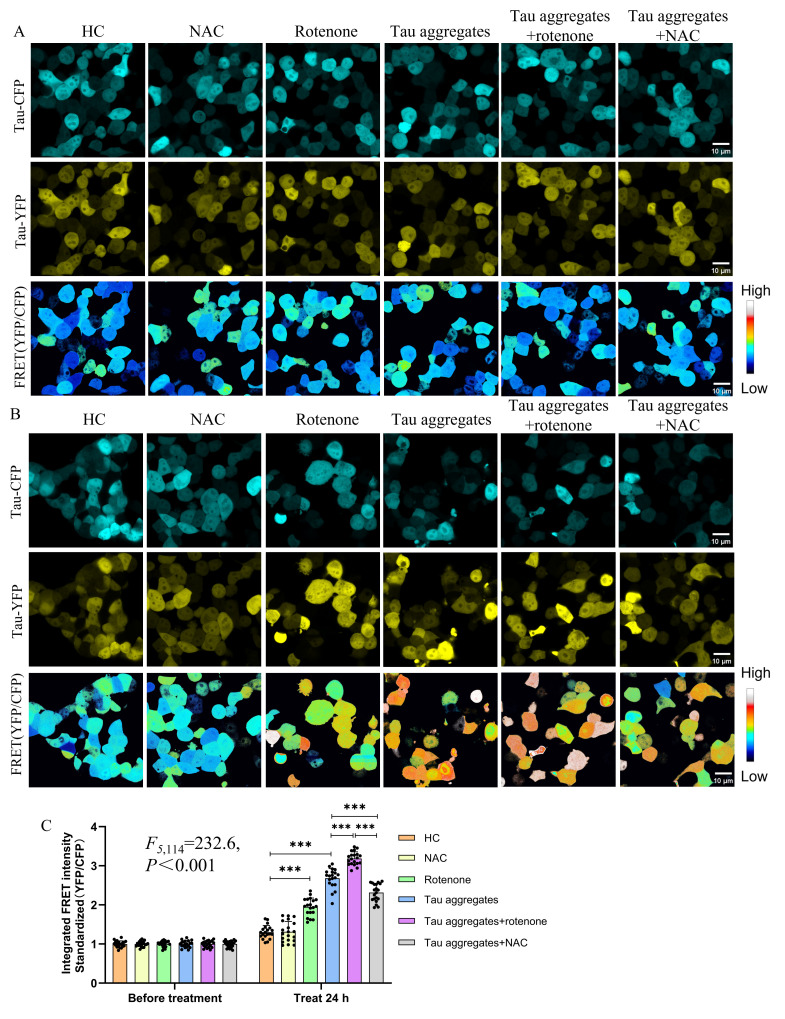
ROS are important risk factors in the process of Tau aggregation in cells. (**A**) Representative confocal micrograph of Tau-ECFP, Tau-EYFP, and FRET intensity in HEK-293T cells before treatment. Scale bar, 10 μm. (**B**) Representative confocal micrograph of Tau-ECFP, Tau-EYFP, and FRET intensity in HEK-293T cells after treatment. Scale bar, 10 μm. (**C**) FRET intensity quantification bar chart for (**A**,**B**). Data represent means ± SD and were analyzed by one-way ANOVA. *** *p* < 0.001.

Next, we explored the effects of ROS on the aggregation of intracellular Tau. We verified the fact that rotenone could promote ROS production and that N-acetylcysteine (NAC) could inhibit ROS production (Figure S4) in order to treat HEK-293T cells with rotenone or NAC for the purposes of promoting or inhibiting ROS production in the cells. In our study, HEK-293T cells expressing both Tau RD/P301S-ECFP and Tau RD/P301S-EYFP were treated with rotenone, NAC, and Tau aggregates for 24 h, after which the FRET intensity was measured. The results showed that there were no significant differences in FRET intensity among the different groups before treatment (Figure 5A,C). The FRET intensity of the HEK-293T cells treated with rotenone was significantly higher than that of the cells in the HC group. Additionally, the FRET intensity of the HEK-293T cells treated with rotenone and Tau aggregates significantly increased compared to that of the cells only treated with the Tau aggregates. Surprisingly, the FRET intensity of the HEK-293T cells treated with NAC and Tau aggregates significantly decreased compared to that of the cells only treated with the Tau aggregates (Figure 5B,C). These results indicate that extracellular Tau aggregates lead to increased ROS levels and that ROS promote intracellular Tau protein aggregation.

### 3.4. ScFv T1 Inhibits the Aggregation of Tau Proteins Both Extracellularly and Intracellularly

**Figure 6:**
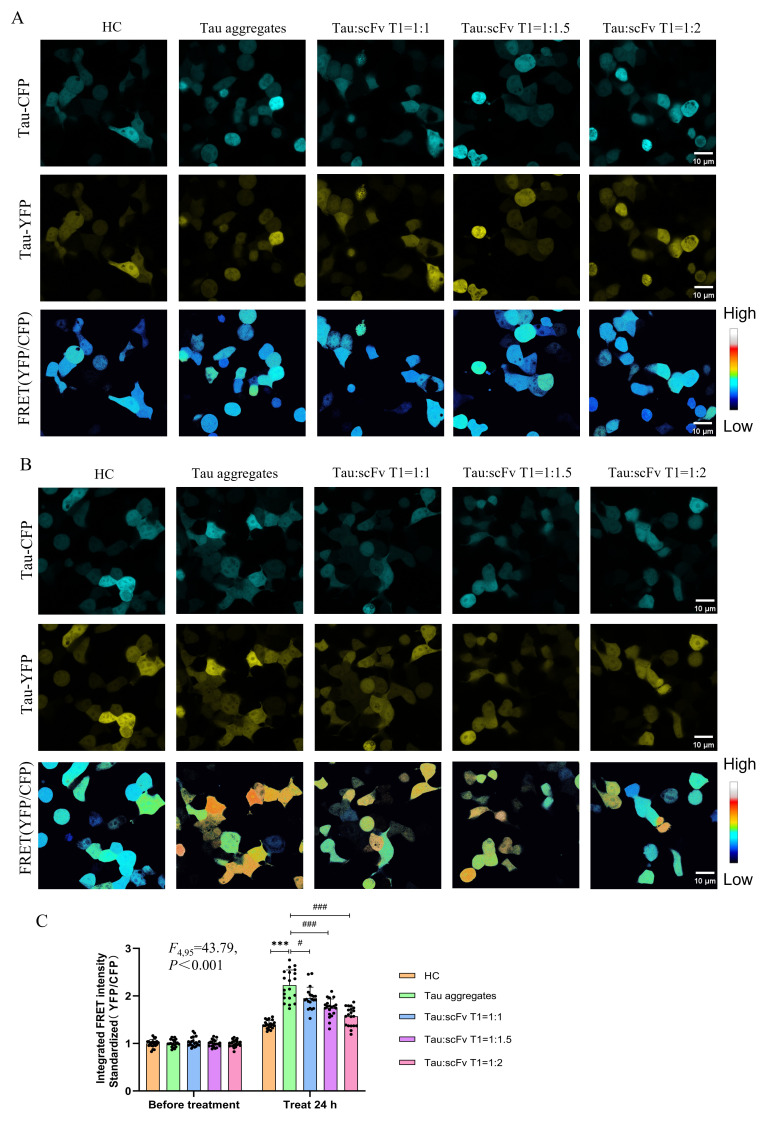
ScFv T1 reduces intracellular Tau aggregation by inhibiting extracellular Tau aggregation. (**A**) Representative confocal micrograph of Tau-ECFP, Tau-EYFP, and FRET intensity in HEK-293T cells before treatment. Scale bar, 10 μm. (**B**) Representative confocal micrograph of Tau-ECFP, Tau-EYFP, and FRET intensity in HEK-293T cells after treatment. Scale bar, 10 μm. (**C**) FRET intensity quantification bar chart for (**A**,**B**). Data represent means ± SD and were analyzed by one-way ANOVA. *** *p* < 0.001, ^#^ *p* < 0.05, ^###^ *p* < 0.001.

Given that Tau protein aggregation and propagation are fundamental causes of the development of AD, antibody therapy targeting Tau proteins has shown great potential for inhibiting the pathological progression of AD. Here, we studied the effects of a previously screened single-chain variable fragment antibody (scFv T1) on the aggregation of Tau proteins. ScFv T1 has been tested and verified to be able to inhibit Tau aggregation in vitro (Figure S5). Tau monomers and scFv T1 were incubated together with different protein ratios (Tau:scFv T1 = 1:0, 1:1, 1:1.5, and 1:2) for 48 h. The recombinant plasmids expressing Tau RD/P301S-ECFP and Tau RD/P301S-EYFP were transfected into HEK-293T cells for 24 h. Next, the cells were treated with Tau aggregates and Tau–scFv T1 mixtures for 24 h, and the FRET intensity of the cells was detected. The results showed that there was no significant difference in FRET intensity among the different groups before treatment (Figure 6A,C). The FRET intensity of the cells treated with the Tau–scFv T1 mixtures decreased in an scFv T1 concentration-dependent manner compared with that of the cells only treated with Tau aggregates (Figure 6B,C). This result indicates that scFv T1 could specifically bind to Tau proteins outside the cell, inhibit the aggregation of these Tau proteins, and prevent their inductive effect on intracellular Tau protein aggregation when used extracellularly.

**Figure 7:**
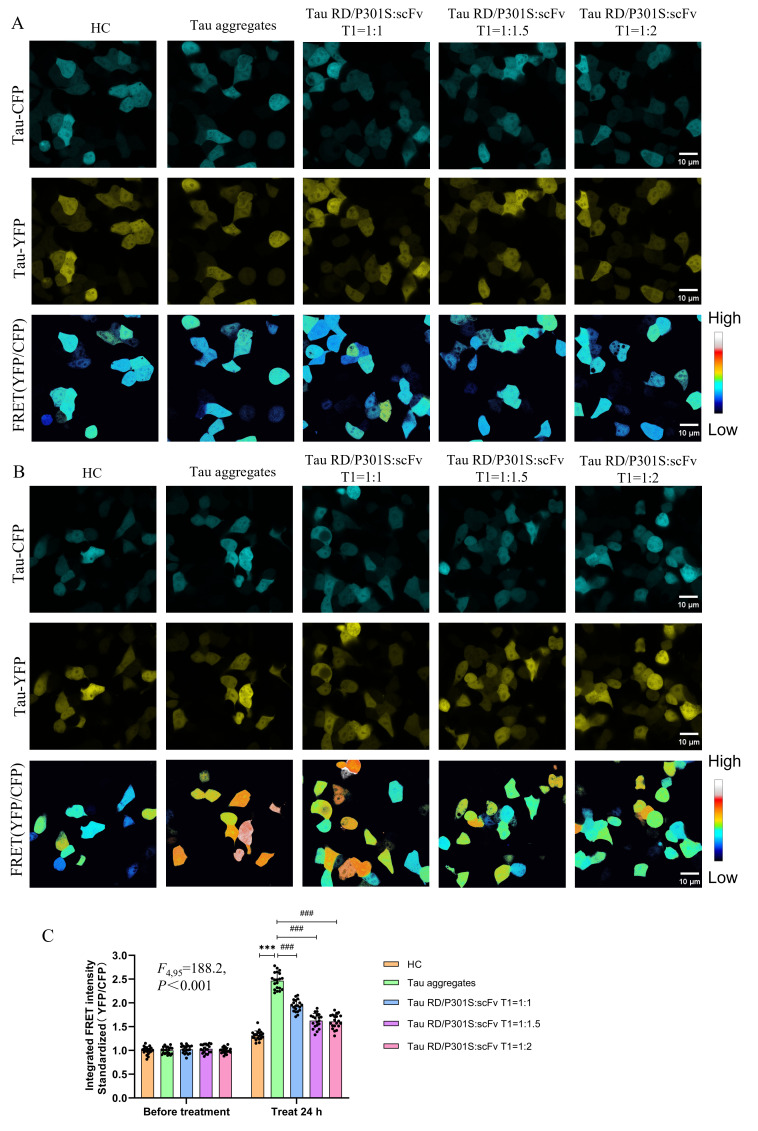
ScFv T1 inhibits the aggregation of Tau protein intracellularly. (**A**) Representative confocal micrograph of Tau-ECFP, Tau-EYFP, and FRET intensity in HEK-293T cells expressing scFv T1 before treatment. Scale bar, 10 μm. (**B**) Representative confocal micrograph of Tau-ECFP, Tau-EYFP, and FRET intensity in HEK-293T cells expressing scFv T1 after treatment. Scale bar, 10 μm. (**C**) FRET intensity quantification bar chart for (**A**,**B**). Data represent means ± SD and were analyzed by one-way ANOVA. *** *p* < 0.001, ^###^ *p* < 0.001.

To further investigate the potential of scFv T1 to inhibit the aggregation of Tau proteins in cells, the recombinant plasmids expressing Tau RD/P301S-ECFP, Tau RD/P301S-EYFP, and scFv T1 were cotransfected into HEK-293T cells at different ratios (Tau RD/P301S-ECFP:Tau RD/P301S-EYFP:scFv T1 = 1:1:0, 1:1:2, 1:1:3, and 1:1:4). ScFv T1 expression was confirmed using Western blot analysis (Figure S6). All cells except those in the heparin treatment control group were treated with Tau aggregates for 24 h, after which FRET intensity was measured. The results showed that the FRET intensity of the cells expressing RD/P301S-ECFP, Tau RD/P301S-EYFP, and scFv T1 significantly decreased in an scFv T1 concentration-dependent manner compared to that of the cells only expressing Tau RD/P301S-ECFP and Tau RD/P301S-EYFP (Figure 7A–C). These findings suggest that scFv Tl could inhibit the intracellular Tau aggregation induced by extracellular Tau aggregates when used intracellularly.

**Figure 8:**
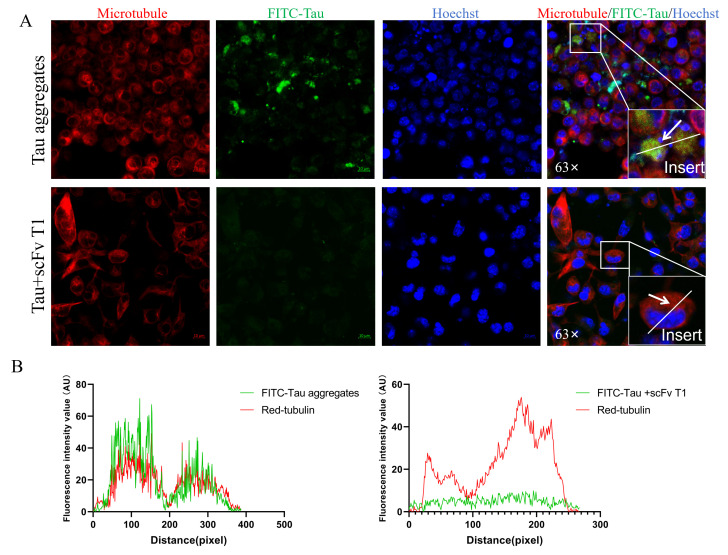
The number of Tau aggregates available for uptake by SH-SY5Y cells decreased after the inhibition of Tau aggregation by scFv T1. (**A**) Representative confocal images showing colocalization of SH-SY5Y tubulin and FITC–Tau. The experiment was performed in triplicate. Red fluorescence: microtubule stained with far-infrared fluorescence staining kit. Green fluorescence: FITC–Tau. Blue fluorescence: nuclei stained with Hoechst 33342. Scale bar, 10 μm. (**B**) Intensity wave showing the colocalization of FITC–Tau and Red-tubulin at the white arrow.

To further investigate the uptake of Tau by SH-SY5Y cells after the extracellular inhibition of Tau aggregation by scFv T1, FITC-modified Tau monomers were incubated with scFv T1 for 48 h. The SH-SY5Y cells were treated with FITC–Tau aggregates or FITC–Tau–scFv T1 mixtures for 24 h and detected using laser confocal microscopy. The results showed that the FITC–Tau was not colocalized to microtubules in the presence of scFv T1 (Figure 8A,B and Figure S7). This indicates that, after scFv T1 inhibited Tau aggregation, the amount of Tau aggregates available for uptake by SH-SY5Y cells was reduced.

**Figure 9:**
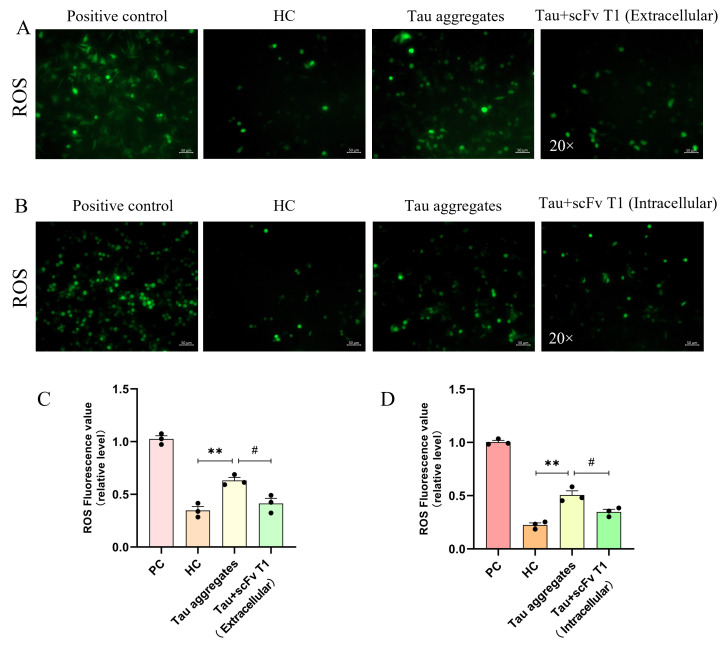
ScFv T1 inhibits the ROS accumulation induced by Tau aggregates both extracellularly and intracellularly. (**A**) Representative fluorescence micrograph showing ROS levels. Tau monomers were incubated with scFv T1 extracellularly. Scale bar, 50 μm. (**B**) Representative fluorescence micrograph showing ROS levels. ScFv T1 was expressed in SH-SY5Y cells. Scale bar, 50 μm. (**C**) Bar chart of the relative ROS levels in (**A**). (**D**) Bar chart of the relative ROS levels in (**B**). Data represent means ± SD and were analyzed by Student’s *t*-test. ** *p* < 0.01, ^#^ *p* < 0.05.

To explore the effect of scFv T1 on Tau aggregates-induced ROS in cells, the Tau proteins were first incubated with scFv T1 for 48 h; then, SH-SY5Y cells were treated with Tau aggregates and Tau–scFv T1 mixtures for 24 h, after which the ROS levels in the cells were detected. The results showed that the ROS levels in the cells treated with the Tau–scFv T1 mixtures decreased compared to those in the cells only treated with Tau aggregates (Figure 9A,C). In addition, we explored the effect of the intracellular scFv T1 on ROS levels. The recombinant plasmid expressing scFv T1 was transfected into SH-SY5Y cells. The cells were treated with Tau aggregates for 24 h, after which the ROS levels in the cells were detected. The results showed that the ROS levels in the cells expressing scFv T1 decreased compared to those in the cells not expressing scFv T1 (Figure 9B,D). These results indicate that scFv T1 could alleviate the ROS accumulation caused by Tau aggregates both extracellularly and intracellularly.

In conclusion, scFv T1 effectively inhibits the aggregation of Tau proteins both extracellularly and intracellularly. It can also reduce the amount of Tau aggregates available for uptake by SH-SY5Y cells and alleviate ROS accumulation in cells caused by extracellular Tau aggregates.

### 3.5. ScFv T1 Alleviates Neurotoxicity Caused by Extracellular Tau Aggregates

**Figure 10:**
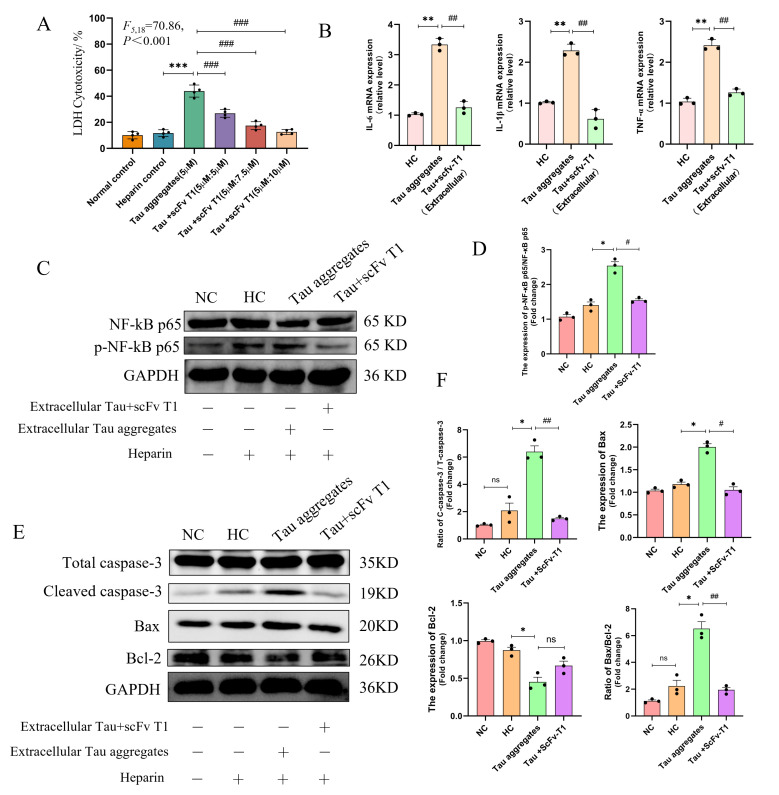
ScFv T1 alleviates inflammation and apoptosis caused by Tau aggregates. (**A**) The cytotoxicity of different samples to SH-SY5Y cells detected by the LDH method. This experiment was repeated four times. (**B**) The mRNA levels of IL-6, IL-1β, and TNF-α detected by qPCR. The experiment was performed in triplicate. (**C**) The protein levels of NF-kB p65 and p-NF-kB p65 detected by Western blot. The grouping of blots cropped from different gels of the same samples. The experiment was performed in triplicate. (**D**) Bar chart of the relative protein expression levels in (**C**). (**E**) The expression levels of Bax, Bcl-2, caspase-3, and cleaved caspase-3 detected by Western blot. The grouping of blots cropped from different gels of the same samples. The experiment was performed in triplicate. (**F**) Bar chart of the relative protein expression levels in (**E**). Data represent means ± SD and were analyzed by one-way ANOVA (**A**) or Student’s *t*-test (**B**,**D**,**F**). * *p* < 0.05, ** *p* < 0.01, *** *p* < 0.001, ^#^ *p* < 0.05, ^##^ *p* < 0.01, ^###^ *p* < 0.001, ns, not significant.

To further explore the effect of scFv T1 on neurotoxicity caused by extracellular Tau aggregates, SH-SY5Y cells were treated with Tau aggregates or Tau–scFv T1 mixtures for 24 h. The results of the LDH assay conducted showed that scFv T1 reduced the cytotoxicity of Tau aggregates to SH-SY5Y in a dose-dependent manner (Figure 10A). Additionally, the mRNA levels of inflammatory cytokines and the protein level of p-NF-kB p65 were detected. The results show that, compared to the Tau aggregate treatment group, the mRNA levels of the inflammatory cytokines IL-6, IL-1β, and TNF-α and the protein level of p-NF-kB p65 decreased in the Tau–scFv T1 treatment group (Figure 10B–D). The expression levels of apoptosis-related proteins were also detected in the absence and presence of scFv T1. The results showed that the levels of Bax and cleaved caspase-3 decreased when scFv T1 was present (Figure 10E,F). These findings suggest that scFv T1 has the potential to prevent and ameliorate Tau aggregates-induced cellular inflammation and apoptosis by inhibiting Tau aggregation.

## 4. Discussion

The process of nerve cell damage caused by Tau aggregates is highly significant in the pathogenesis of AD [28], but the toxic effects of extracellular Tau aggregates on nerve cells and their related mechanisms remain unclear. Endogenous Tau is likely released into the extracellular space upon neuron degeneration, where it can then be toxic to other neurons [29]. In our experiments, we prepared Tau aggregates by incubating Tau monomers with heparin for 48 h. To verify the damaging effect of the extracellular Tau aggregates on nerve cells, a nerve cell model treated with extracellular Tau aggregates was established. The extracellular Tau aggregates induced via heparin were found to be toxic to the SH-SY5Y cells used in this study.

Inflammation is increasingly acknowledged as a key factor in the pathogenesis of AD. Neuronal loss and inflammation can be caused by Tau protein overexpression and aggregation, as was demonstrated in mice with tauopathy [30]. Moreover, activation of the NF-κB signaling pathway was found to mediate the spread of Tau in young PS19 mice and further lead to neuroinflammation [31]. In our study, after SH-SY5Y cells were treated with Tau aggregates for 24 h, the NF-κB signaling pathway was activated while the mRNA levels of the inflammatory factors IL-6, IL-1β, and TNF-α increased, suggesting that the extracellular Tau aggregates stimulated an inflammatory response in the SH-SY5Y cells. Sustained inflammation often induces cell damage and even apoptosis [32]. Tau deposition can trigger apoptotic pathways that result in neuronal death [33]. Tau aggregates have been discovered to cause microglia pyroptosis through the NLRP3-ASC axis, inducing oxidative stress and caspase-dependent apoptosis [34]. In our study, we found that extracellular Tau aggregates caused an increase in the ratio of Bax to bcl-2, together with an increase in the expression level of cleaved caspase-3 in SH-SY5Y cells, indicating increased apoptosis.

Tau aggregation and propagation have attracted much attention in the progression of AD after the Tau seeding hypothesis was proposed [35]. Misfolded or pathological conformers of Tau undergo cell-to-cell spread in a tauopathy strain-specific manner [36]. Pathological Tau is a type of aggregate that can spread between neurons, and it has been proposed that extracellular Tau proteins enter human neurons via endocytosis and exosomes [37,38]. To verify this theory, SH-SY5Y cells were treated with FITC-modified Tau aggregates, and FITC–Tau was found to be colocalized to the microtubule cytoskeleton in the cells. Thus, we agree with the theory that Tau proteins enter neurons. Additionally, to further explore the effect of extracellular Tau aggregates on intracellular Tau aggregation, we constructed recombinant plasmids expressing Tau RD/P301S-ECFP and Tau RD/P301S-EYFP for FRET experiments. These plasmids were transfected into HEK-293T cells without the endogenous Tau proteins, and the degree of Tau RD/P301S aggregation was determined via FRET intensity (the ratio of EYFP/ECFP intensity). We found that extracellular Tau aggregates induced the aggregation of intracellular Tau RD/P301S monomers. Overall, this research shows that extracellular Tau aggregates can enter SH-SY5Y cells and promote Tau aggregation in them.

In cell models induced via Tau aggregates, ROS are accumulated. ROS are important due to their upstream and downstream effects on tauopathy, and there is clear evidence that ROS directly promote Tau modifications in tauopathy [39,40]. In this study, we found that extracellular Tau aggregates induced the accumulation of ROS. Additionally, to explore the effect of ROS on Tau aggregation, HEK-293T cells were treated with NAC or rotenone. Rotenone promoted ROS production in the cells and intracellular Tau aggregation, whereas NAC inhibited intracellular Tau aggregation induced by extracellular Tau aggregates. Therefore, we conclude that ROS play an important role in intracellular Tau aggregation. Moreover, Xiongwei Zhu et al. reported that oxidative-stress-mediated ROS production is involved in protein oxidation and forms stable, advanced end products, and these protein products are evident in NFTs in AD [41]. In this study, we only detected ROS levels and their relationship to Tau aggregation in cells and did not engage in a detailed study of the related mechanisms by which ROS promote Tau aggregation. This may be the focus of future research.

The seeding and toxicity of Tau aggregates are particularly important in the progression of AD [42], with anti-Tau propagation immunotherapy becoming an area of interest for therapeutic development against AD. In line with this, we believe that antibody therapies targeting Tau are highly promising. The single-chain variable fragment antibody (ScFv T1) that we screened can specifically bind Tau proteins and inhibit Tau aggregation in vitro [16]. In this study, we explored the inhibitory effect of scFv T1 on Tau aggregation and the cytotoxicity of Tau aggregates, discovering that fewer Tau aggregates are applied to cells for uptake after scFv T1 inhibits Tau aggregation. ScFv T1 can inhibit ROS accumulation and intracellularly Tau aggregation induced by extracellular Tau aggregates both extracellularly and intracellularly. ScFv T1 additionally prevents the aggregation of Tau proteins within cells and outside cells, and Tau–scFv T1 mixtures are not significantly toxic to SH-SY5Y cells. Moreover, scFv T1 alleviates the inflammation and apoptosis caused by Tau aggregation.

Although this study provided important insights into the toxic effects of extracellular Tau aggregates on neurons and the inhibitory effects of Tau-targeting antibodies on Tau aggregation and toxicity, we did not explore them through in vivo experiments, and we intend to explore this in future research efforts. In addition, we used heparin-induced Tau aggregates; however, the structure of the Tau aggregates induced in vitro via heparin in this study may be different from that in patients with AD, which is also a limitation of this study. In the future, we will use disease-related materials to conduct research on the toxicity and mechanism of Tau aggregates.

## 5. Conclusions

In conclusion, extracellular Tau aggregates cause intracellular Tau aggregation after entering cells, and ROS promotes this process. Moreover, scFv T1 inhibits the accumulation of ROS and alleviates the inflammation and apoptosis induced by Tau aggregates by inhibiting the aggregation of Tau. Our findings could provide new evidence for the study of neurotoxicity and related mechanisms involving extracellular Tau aggregates in addition to insights into the development of novel therapeutic agents for the treatment and prevention of AD.

## Data Availability

All data generated or analyzed during this study are included in this published article (and its Supplementary Information files).

## Supplementary Materials

The following supporting information can be downloaded at: https://www.mdpi.com/article/10.3390/biom15060872/s1, Figure S1: Tau monomers were induced to aggregate by heparin. ThT fluorescence intensity was measured at different time intervals. This experiment was repeated four times. The data are presented as mean ± SD; Figure S2: Measurement of the cytotoxicity of Tau aggregates to SH-SY5Y cells and HEK-293T cells; Figure S3: Extracellular Tau aggregates enter SH-SY5Y cells. Representative confocal images including multiple z-stacks showing colocalization of SH-SY5Y tubulin and FITC-Tau (Corresponding to Figure 2). Red fluorescence: microtubule stained with far-infrared fluorescence staining kit; Green fluorescence: FITC-Tau; Blue fluorescence: nuclei stained with Hoechst 33342. Scale bar, 10 μm; Figure S4: Rotenone activates ROS production, and NAC inhibits ROS production; Figure S5: ScFv T1 inhibits extracellular Tau aggregation. Tau monomers were incubated with scFv T1 of different concentrations in the aggregation buffer. ThT fluorescence intensity was measured at different time intervals. This experiment was repeated four times. The data are presented as mean ± SD; Figure S6: ScFv T1 was expressed in HEK-293T cells. The recombinant plasmids expressing Tau RD/P301S-ECFP, Tau RD/P301S-EYFP and scFv T1-FLAG were co-transfected into HEK-293T cells at different ratios (Tau RD/P301S-ECFP:Tau RD/P301S-EYFP:scFv T1= 1:1:0, 1:1:2, 1:1:3 and 1:1:4). The expression level of scFv T1-FLAG was measured by western blot. The grouping of blots cropped from different gels of same samples; Figure S7: The number of Tau aggregates available for uptake by SH-SY5Y cells decreased after the inhibition of Tau aggregation by ScFv T1. Representative confocal images including multiple z-stacks showing colocalization of SH-SY5Y tubulin and FITC-Tau (Corresponding to Figure 8). Red fluorescence: microtubule stained with far-infrared fluorescence staining kit; Green fluorescence: FITC-Tau; Blue fluorescence: nuclei stained with Hoechst 33342. Scale bar, 10 μm. And Western Blot Original Images.
