# Supplementary material for: A Single-Chain Variable Fragment Antibody Alleviates Inflammation and Apoptosis of Neurons by Inhibiting Tau Aggregation
Source: Biomolecules. 2025 Jun 15;15(6):872. doi: 10.3390/biom15060872 (PMC12190225; doi:10.3390/biom15060872)
Supplement: Supplementary file 1 [file biomolecules-15-00872-s001.zip › biomolecules-3601304-Original western blot.pdf]

Original western blot for **“A single-chain variable fragment antibody alleviates inflammation and apoptosis of neurons by inhibiting Tau aggregation”**

The original image corresponds to Figure 1C:

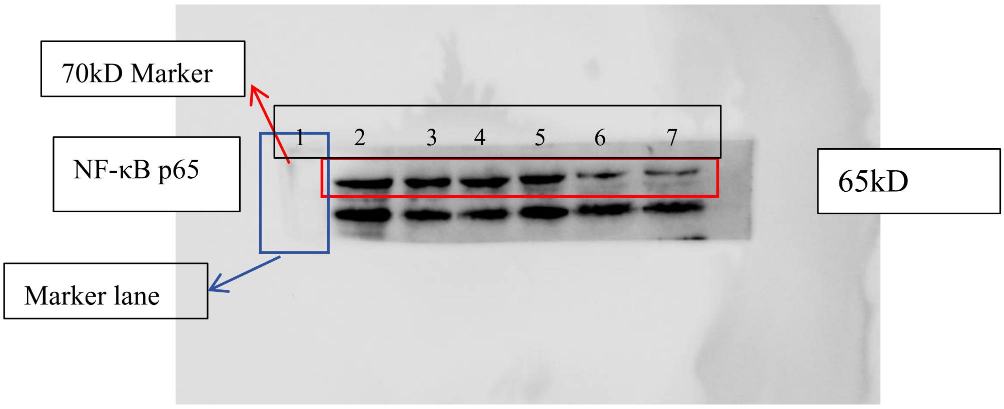

(For Figure 1C in the manuscripts. 1: Marker; 2-3: NC group; 4-5: HC group; 6-7: Tau aggregates group)

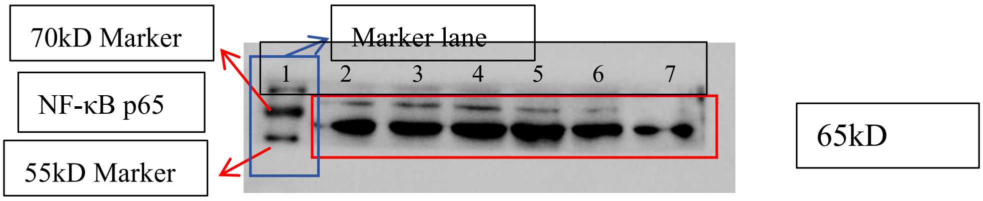

(For quantitative analyses, the result of analysis is shown in Figure 1D. 1: Marker; 2-3: NC group; 4-5: HC group; 6-7: Tau aggregates group)

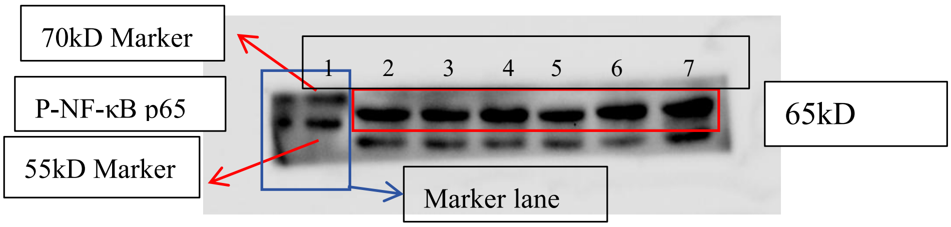

(For Figure 1C in the manuscripts. 1: Marker; 2-3: NC group; 4-5: HC group; 6-7: Tau aggregates group)

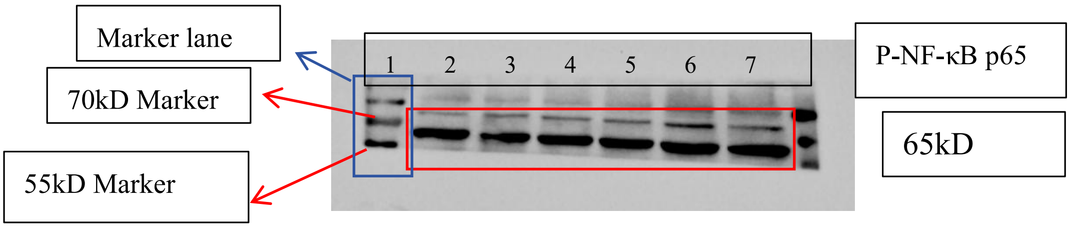

(For quantitative analyses, the result of analysis is shown in Figure 1D. 1: Marker; 2-3: NC group; 4-5: HC group; 6-7: Tau aggregates group)

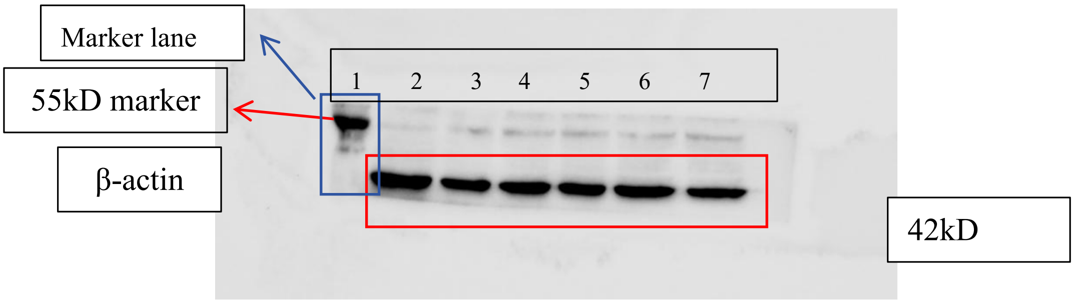

(For Figure 1C in the manuscripts. 1: Marker; 2-3: NC group; 4-5: HC group; 6-7: Tau aggregates group)

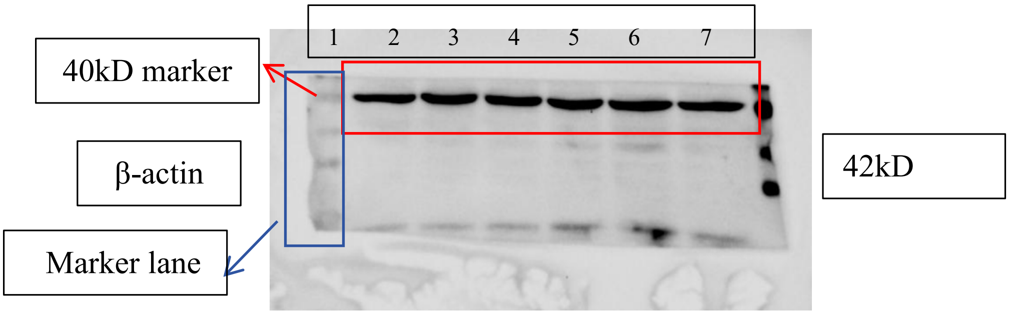

(For quantitative analyses, the result of analysis is shown in Figure 1D. 1: Marker; 2-3: NC group; 4-5: HC group; 6-7: Tau aggregates group)

The original image corresponds to Figure 1E:

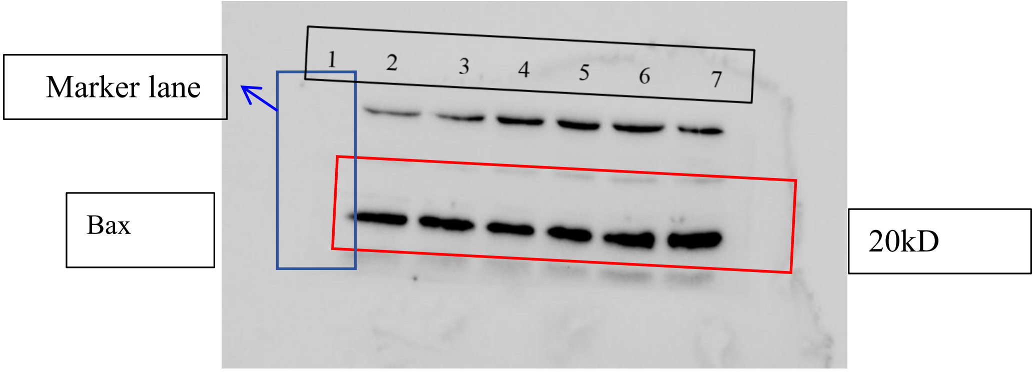

(For Figure 1E in the manuscripts. 1: Marker; 2-3: NC group; 4-5: HC group; 6-7: Tau aggregates group)

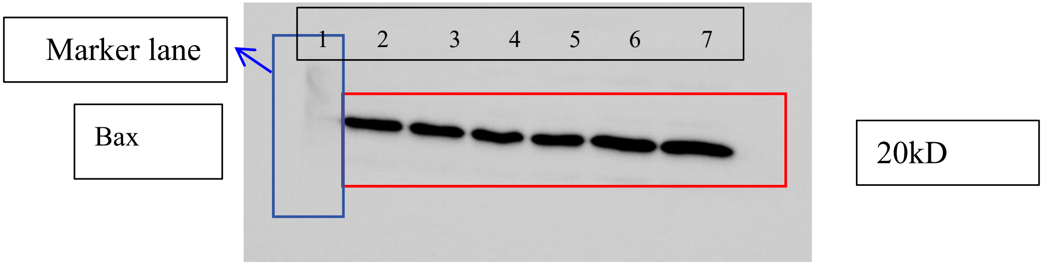

(For quantitative analyses, the result of analysis is shown in Figure 1F. 1: Marker; 2-3: NC group; 4-5: HC group; 6-7: Tau aggregates group)

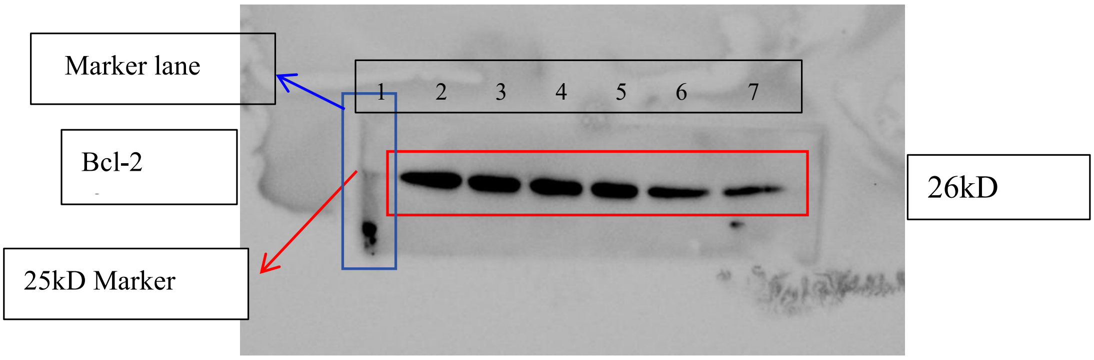

(For Figure 1E in the manuscripts. 1: Marker; 2-3: NC group; 4-5: HC group; 6-7: Tau aggregates group)

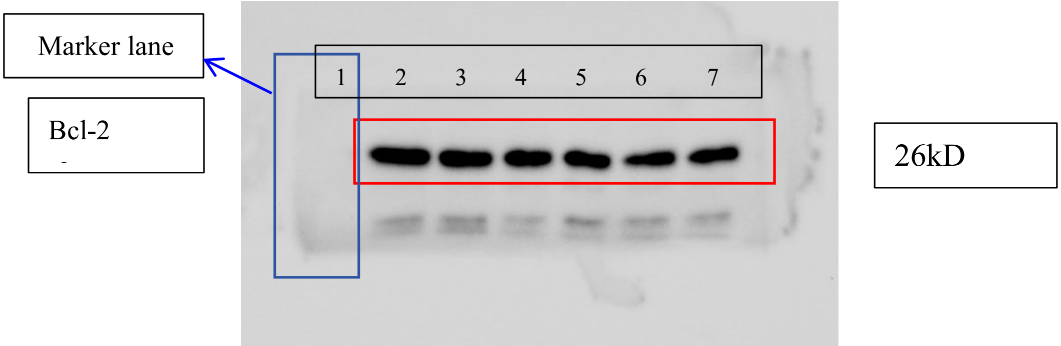

(For quantitative analyses, the result of analysis is shown in Figure 1F. 1: Marker; 2-3: NC group; 4-5: HC group; 6-7: Tau aggregates group)

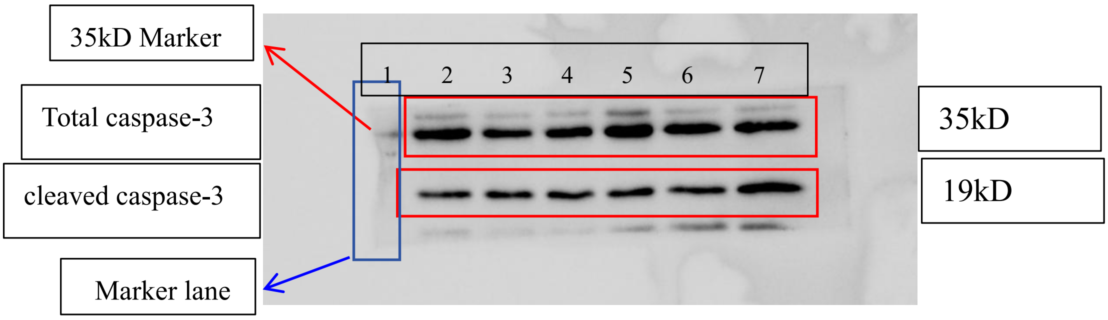

(For Figure 1E in the manuscripts. 1: Marker; 2-3: NC group; 4-5: HC group; 6-7: Tau aggregates group)

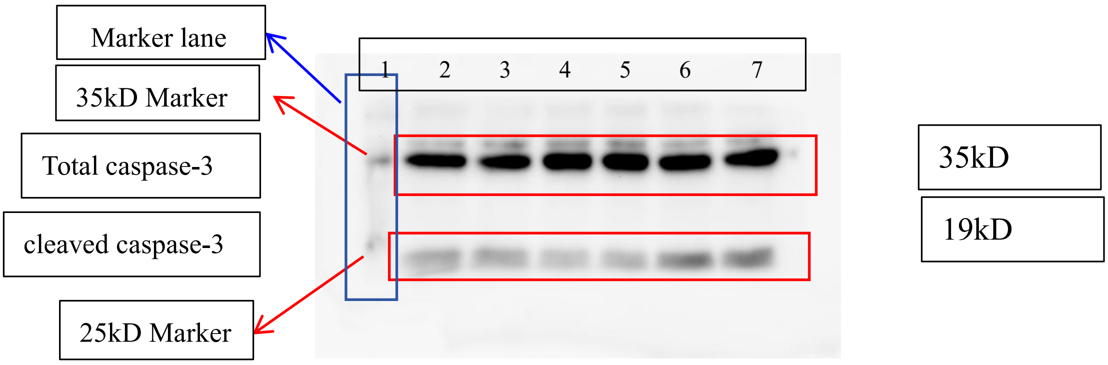

(For quantitative analyses, the result of analysis is shown in Figure 1F. 1: Marker; 2-3: NC group; 4-5: HC group; 6-7: Tau aggregates group)

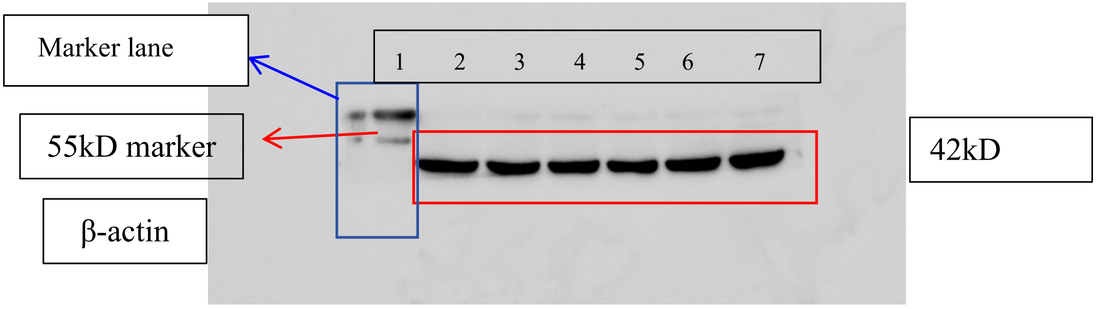

(For Figure 1E in the manuscripts. 1: Marker; 2-3: NC group; 4-5: HC group; 6-7: Tau aggregates group)

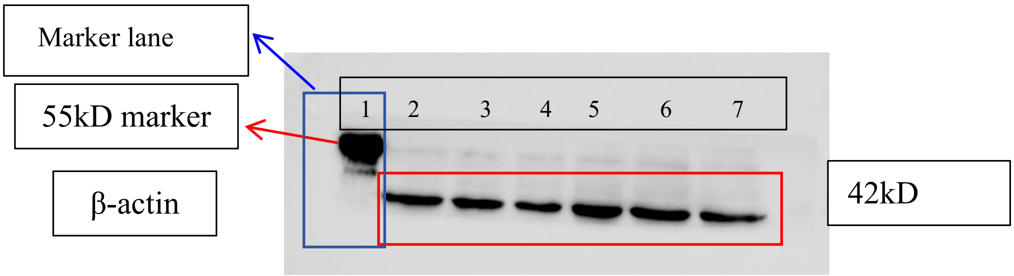

(For quantitative analyses, the result of analysis is shown in Figure 1F. 1: Marker; 2-3: NC group; 4-5: HC group; 6-7: Tau aggregates group)

The original image corresponds to Figure 3D:

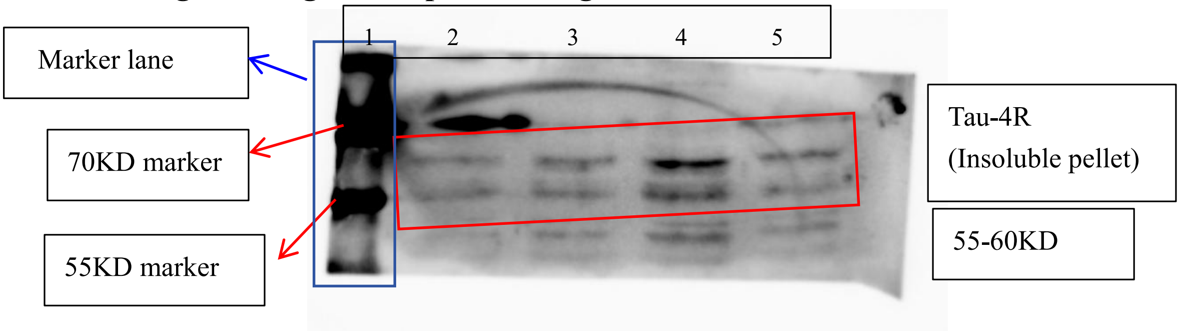

(For Figure 3D in the manuscripts. 1: Marker; 2: NC group; 3: HC group; 4: Tau aggregates group; 5: Tau monomers group)

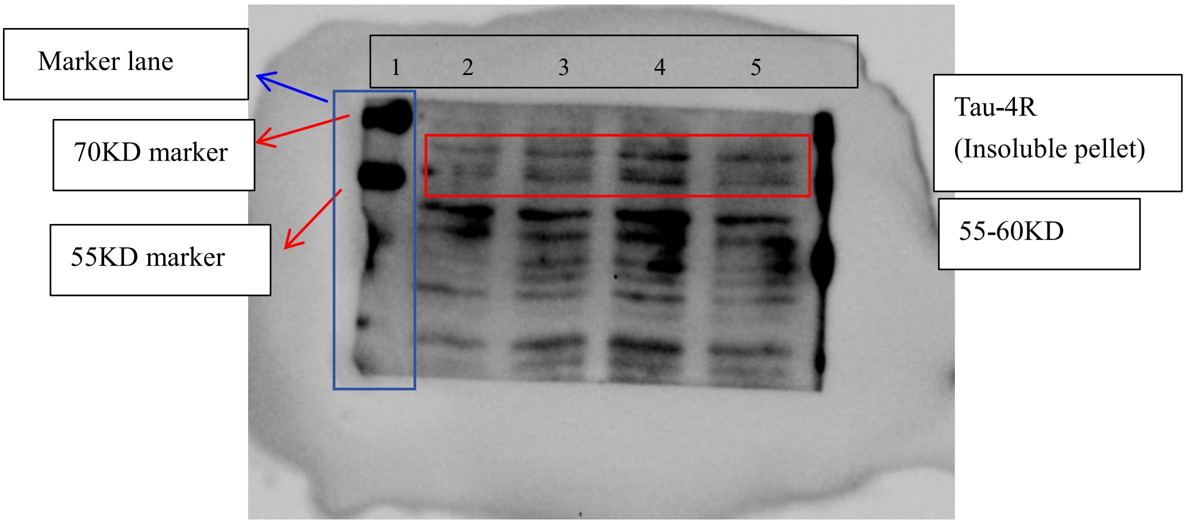

(For quantitative analyses, the result of analysis is shown in Figure 3E. 1: Marker; 2: NC group; 3: HC group; 4: Tau aggregates group; 5: Tau monomers group)

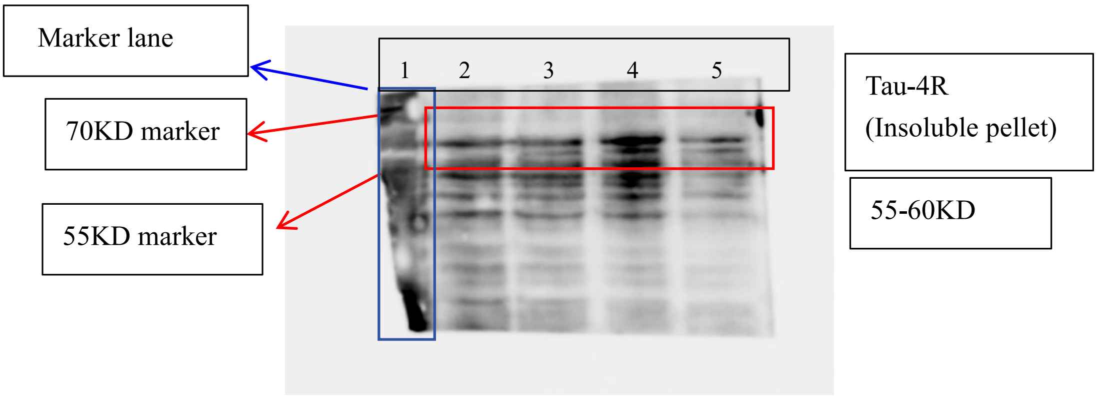

(For quantitative analyses, the result of analysis is shown in Figure 3E. 1: Marker; 2: NC group; 3: HC group; 4: Tau aggregates group; 5: Tau monomers group)

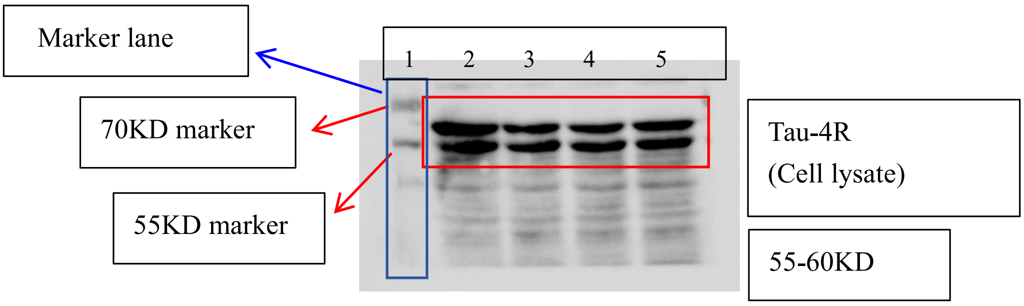

(For Figure 3D in the manuscripts. 1: Marker; 2: NC group; 3: HC group; 4: Tau aggregates group; 5: Tau monomers group)

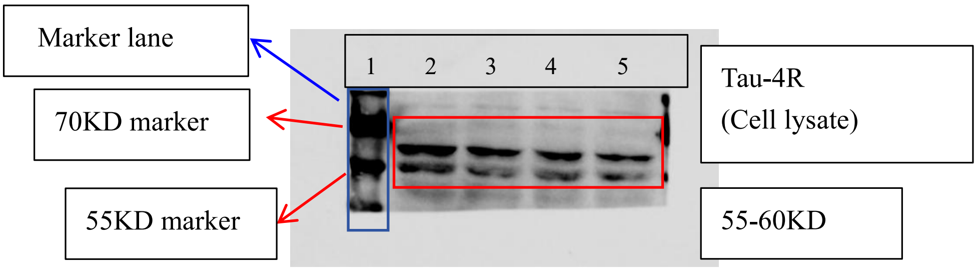

(For quantitative analyses, the result of analysis is shown in Figure 3E. 1: Marker; 2: NC group; 3: HC group; 4: Tau aggregates group; 5: Tau monomers group)

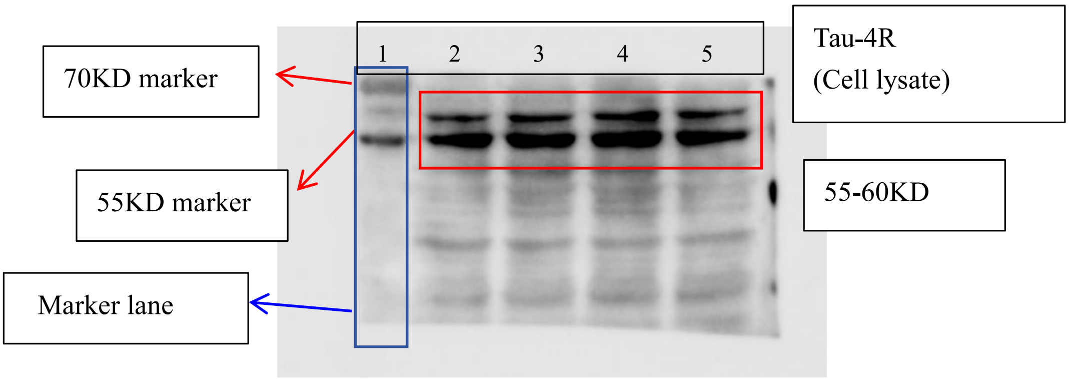

(For quantitative analyses, the result of analysis is shown in Figure 3E. 1: Marker; 2: NC group; 3: HC group; 4: Tau aggregates group; 5: Tau monomers group)

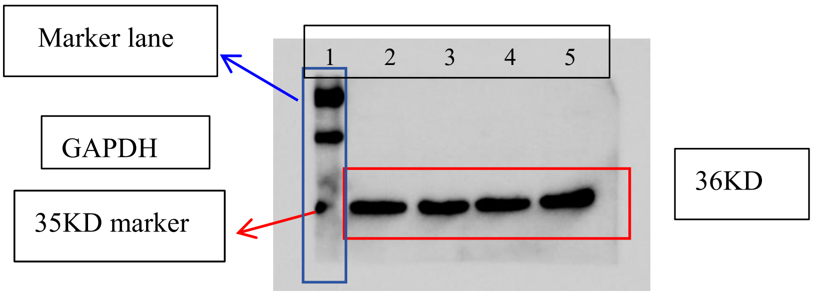

(For Figure 3D in the manuscripts. 1: Marker; 2: NC group; 3: HC group; 4: Tau aggregates group; 5: Tau monomers group)

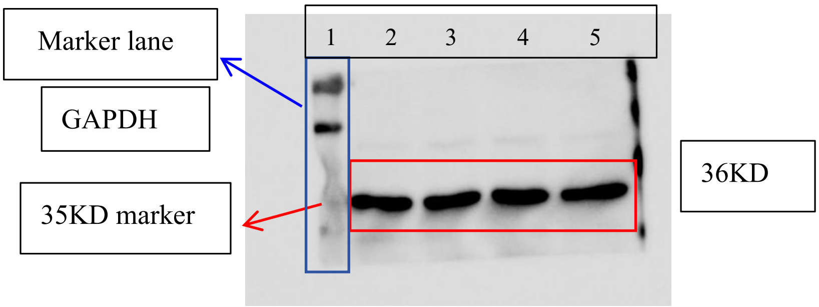

(For quantitative analyses, the result of analysis is shown in Figure 3E. 1: Marker; 2: NC group; 3: HC group; 4: Tau aggregates group; 5: Tau monomers group)

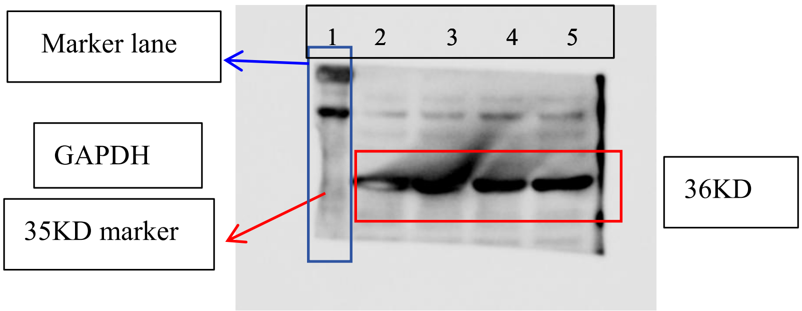

(For quantitative analyses, the result of analysis is shown in Figure 3E. 1: Marker; 2: NC group; 3: HC group; 4: Tau aggregates group; 5: Tau monomers group)

The original image corresponds to Figure 10C:

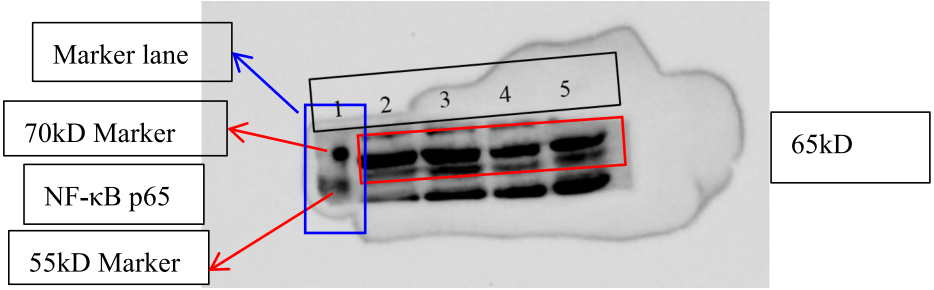

(For Figure 10C in the manuscripts. 1: Marker; 2: NC group; 3: HC group; 4: Tau aggregates group; 5: Tau + scFv T1 group)

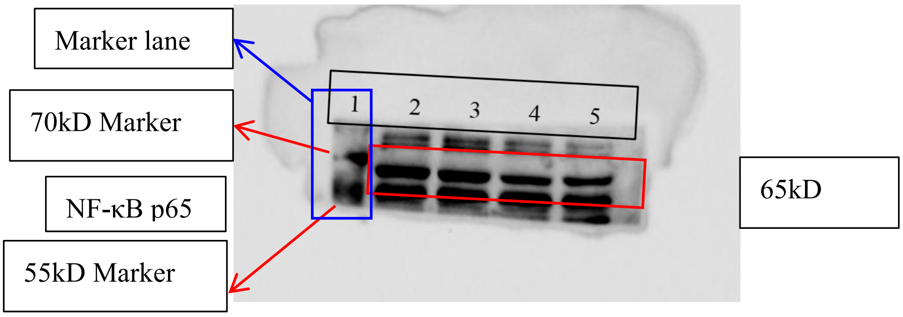

(For quantitative analyses, the result of analysis is shown in Figure 10D. 1: Marker; 2: NC group; 3: HC group; 4: Tau aggregates group; 5: Tau + scFv T1 group)

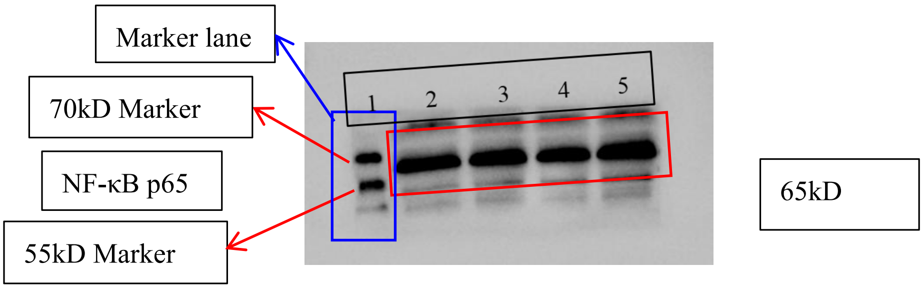

(For quantitative analyses, the result of analysis is shown in Figure 10D. 1: Marker; 2: NC group; 3: HC group; 4: Tau aggregates group; 5: Tau + scFv T1 group)

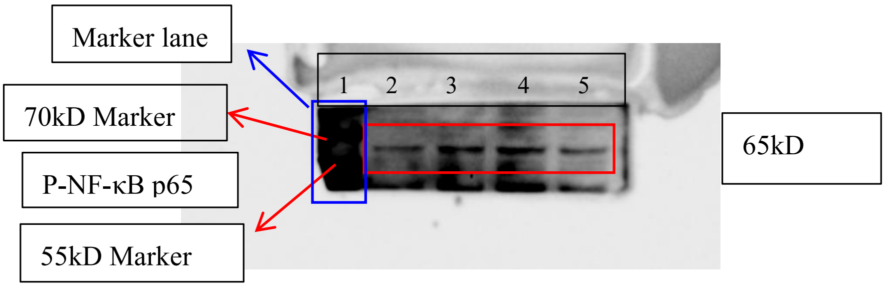

(For Figure 10C in the manuscripts. 1: Marker; 2: NC group; 3: HC group; 4: Tau aggregates group; 5: Tau + scFv T1 group)

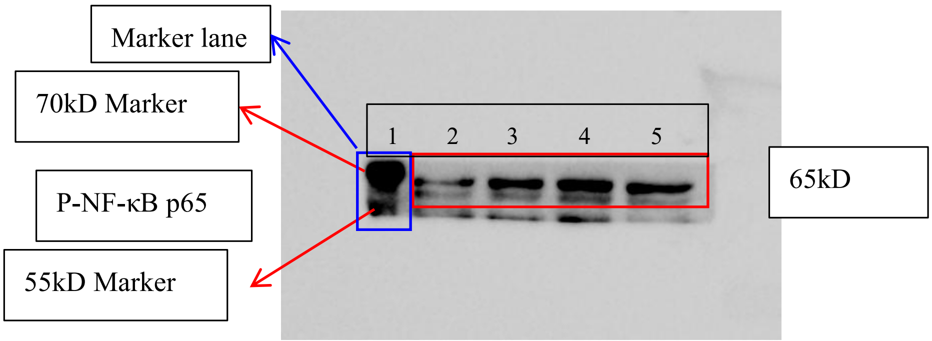

(For quantitative analyses, the result of analysis is shown in Figure 10D. 1: Marker; 2: NC group; 3: HC group; 4: Tau aggregates group; 5: Tau + scFv T1 group)

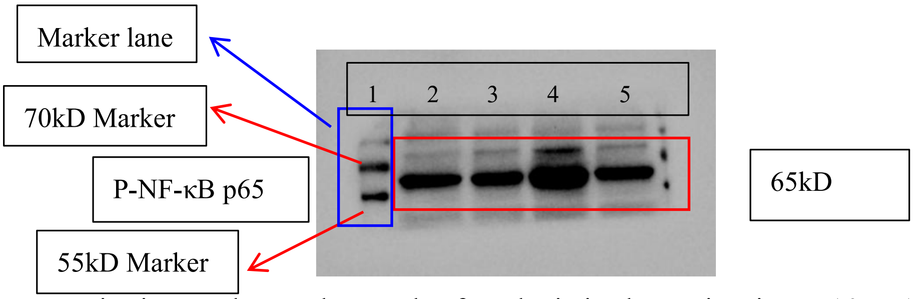

(For quantitative analyses, the result of analysis is shown in Figure 10D. 1: Marker; 2: NC group; 3: HC group; 4: Tau aggregates group; 5: Tau + scFv T1 group)

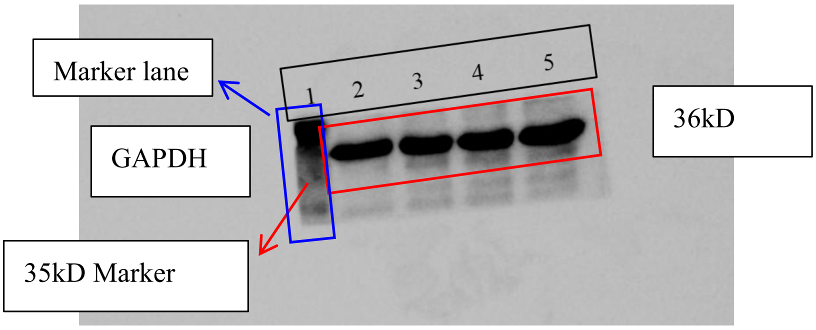

(For Figure 10C in the manuscripts. 1: Marker; 2: NC group; 3: HC group; 4: Tau aggregates group; 5: Tau + scFv T1 group)

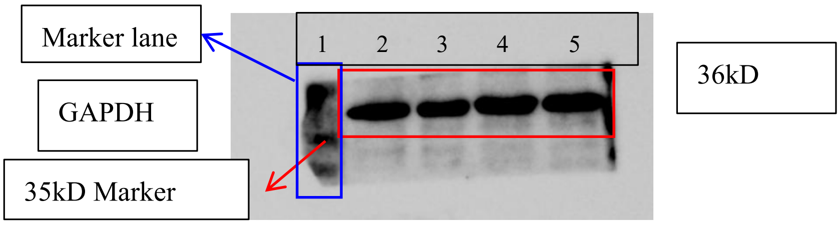

(For quantitative analyses, the result of analysis is shown in Figure 10D. 1: Marker; 2: NC group; 3: HC group; 4: Tau aggregates group; 5: Tau + scFv T1 group)

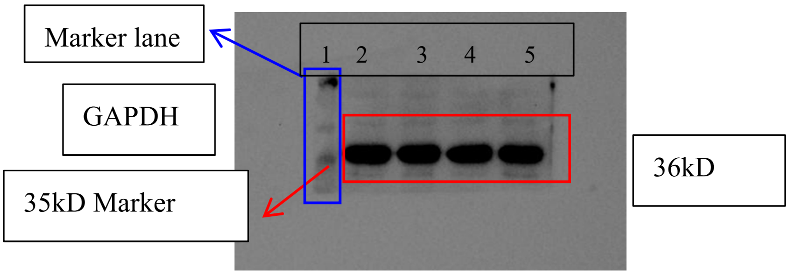

(For quantitative analyses, the result of analysis is shown in Figure 10D. 1: Marker; 2: NC group; 3: HC group; 4: Tau aggregates group; 5: Tau + scFv T1 group)

The original image corresponds to Figure 10E:

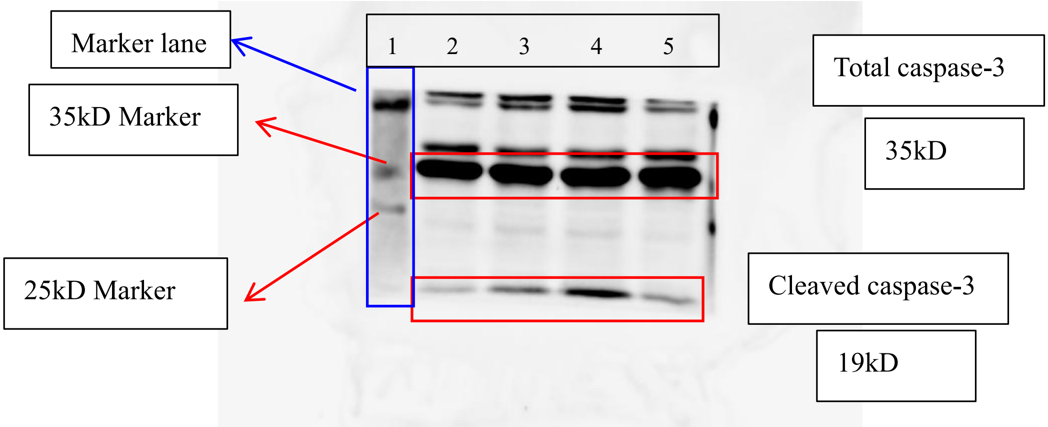

(For Figure 10E in the manuscripts. 1: Marker; 2: NC group; 3: HC group; 4: Tau aggregates group; 5: Tau + scFv T1 group)

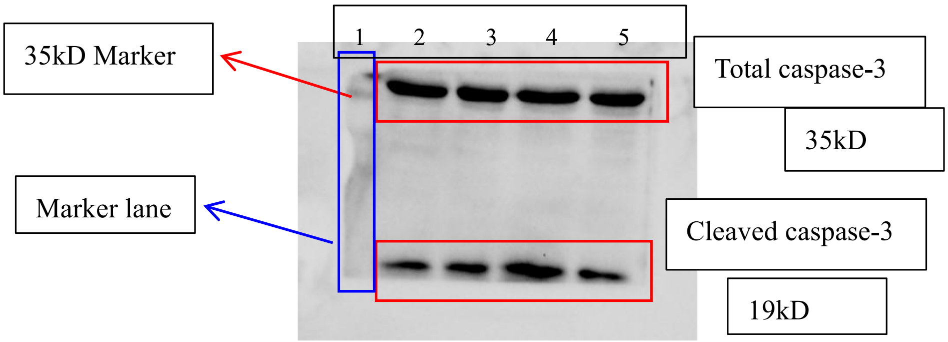

(For quantitative analyses, the result of analysis is shown in Figure 10F. 1: Marker; 2: NC group; 3: HC group; 4: Tau aggregates group; 5: Tau + scFv T1 group)

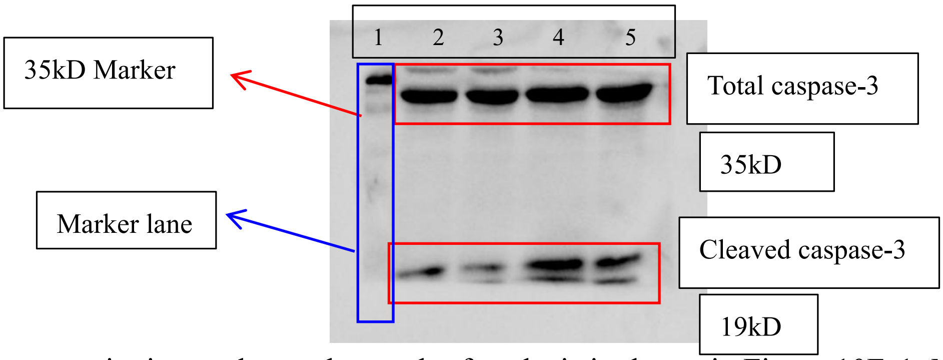

(For quantitative analyses, the result of analysis is shown in Figure 10F. 1: Marker; 2: NC group; 3: HC group; 4: Tau aggregates group; 5: Tau + scFv T1 group)

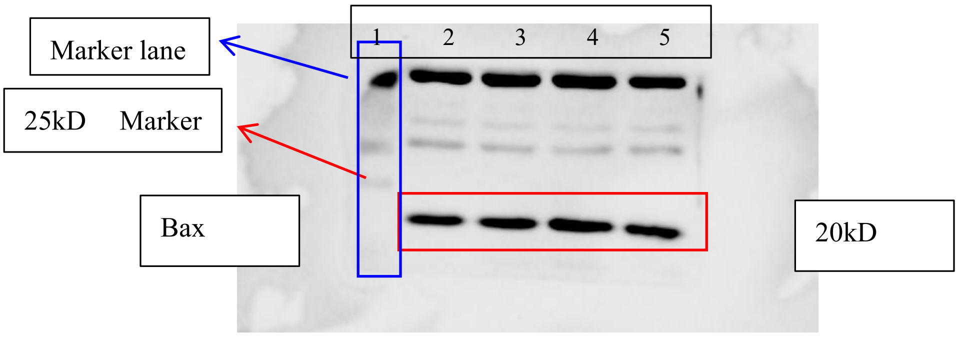

(For Figure 10E in the manuscripts. 1: Marker; 2: NC group; 3: HC group; 4: Tau aggregates group; 5: Tau + scFv T1 group)

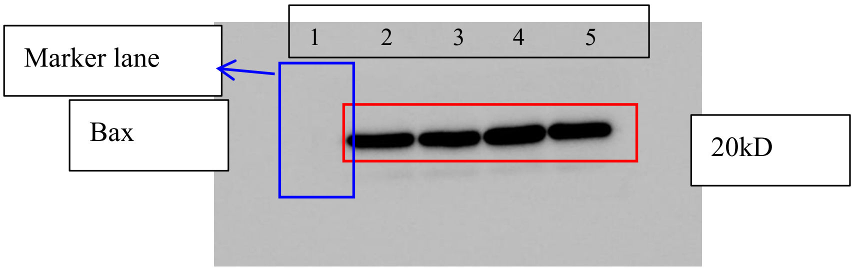

(For quantitative analyses, the result of analysis is shown in Figure 10F. 1: Marker; 2: NC group; 3: HC group; 4: Tau aggregates group; 5: Tau + scFv T1 group)

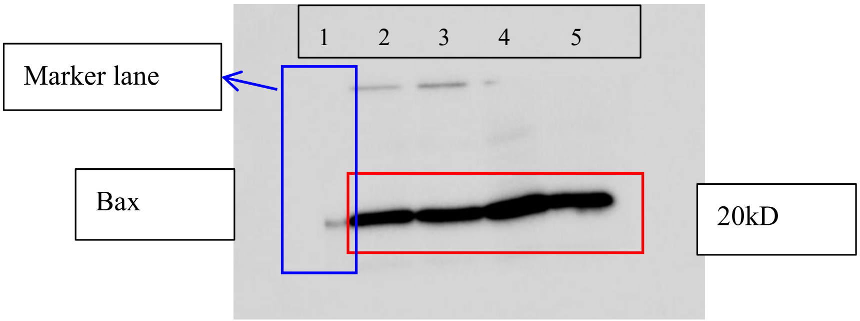

(For quantitative analyses, the result of analysis is shown in Figure 10F. 1: Marker; 2: NC group; 3: HC group; 4: Tau aggregates group; 5: Tau + scFv T1 group)

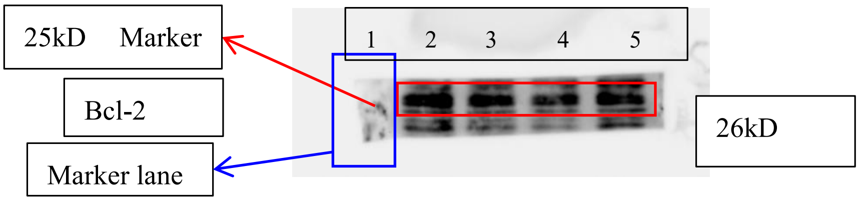

(For Figure 10E in the manuscripts. 1: Marker; 2: NC group; 3: HC group; 4: Tau aggregates group; 5: Tau + scFv T1 group)

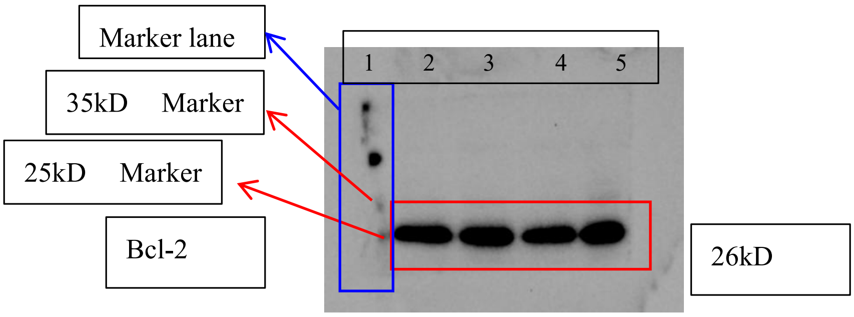

(For quantitative analyses, the result of analysis is shown in Figure 10F. 1: Marker; 2: NC group; 3: HC group; 4: Tau aggregates group; 5: Tau + scFv T1 group)

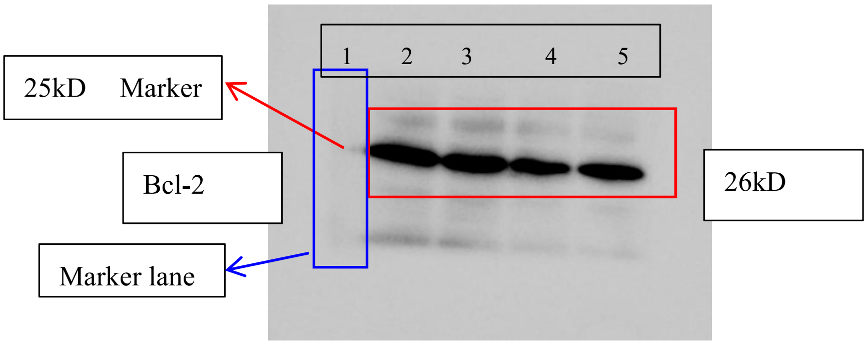

(For quantitative analyses, the result of analysis is shown in Figure 10F. 1: Marker; 2: NC group; 3: HC group; 4: Tau aggregates group; 5: Tau + scFv T1 group)

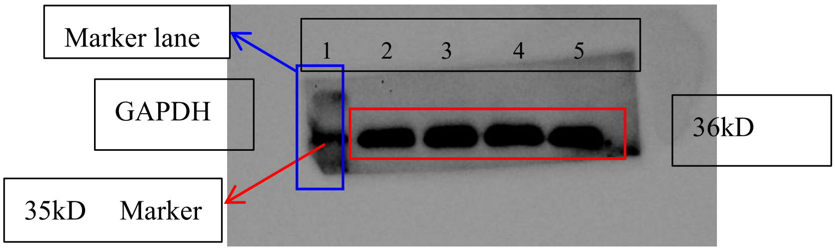

(For Figure 10E in the manuscripts. 1: Marker; 2: NC group; 3: HC group; 4: Tau aggregates group; 5: Tau + scFv T1 group)

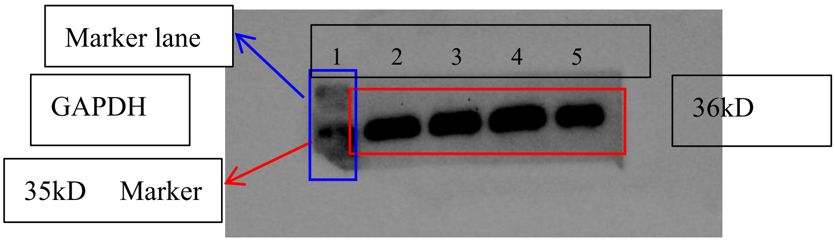

(For quantitative analyses, the result of analysis is shown in Figure 10F. 1: Marker; 2: NC group; 3: HC group; 4: Tau aggregates group; 5: Tau + scFv T1 group)

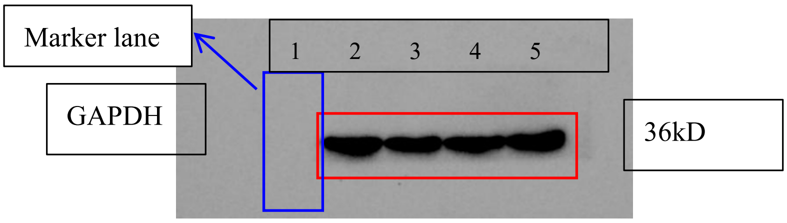

(For quantitative analyses, the result of analysis is shown in Figure 10F. 1: Marker; 2: NC group; 3: HC group; 4: Tau aggregates group; 5: Tau + scFv T1 group)

The original image corresponds to Figure S6:

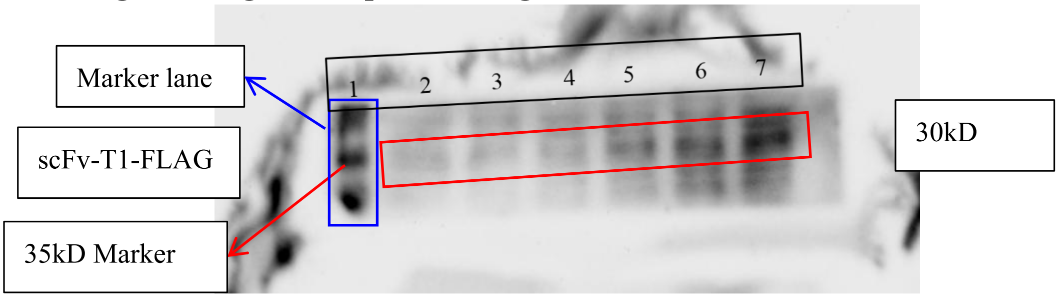

(For Figure S6 in the manuscripts. 1: Marker; 2: NC group; 3: HC group; 4: Tau aggregates group; 5: Tau + scFv T1=1:1 group; 6: Tau + scFv T1=1:1.5 group; 7: Tau + scFv T1=1:2 group)

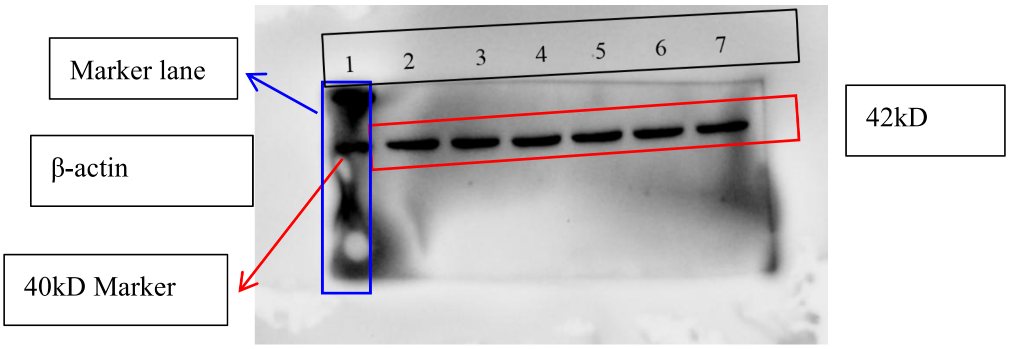

(For Figure S6 in the manuscripts. 1: Marker; 2: NC group; 3: HC group; 4: Tau aggregates group; 5: Tau + scFv T1=1:1 group; 6: Tau + scFv T1=1:1.5 group; 7: Tau + scFv T1=1:2 group)
