# Supplementary material for: A Single-Chain Variable Fragment Antibody Alleviates Inflammation and Apoptosis of Neurons by Inhibiting Tau Aggregation
Source: Biomolecules. 2025 Jun 15;15(6):872. doi: 10.3390/biom15060872 (PMC12190225; doi:10.3390/biom15060872)
Supplement: Supplementary file 1 [file biomolecules-15-00872-s001.zip › biomolecules-3601304-supplementary.pdf]

Supplemental Material for “**A single-chain variable fragment antibody alleviates inflammation and apoptosis of neurons by inhibiting Tau aggregation**”

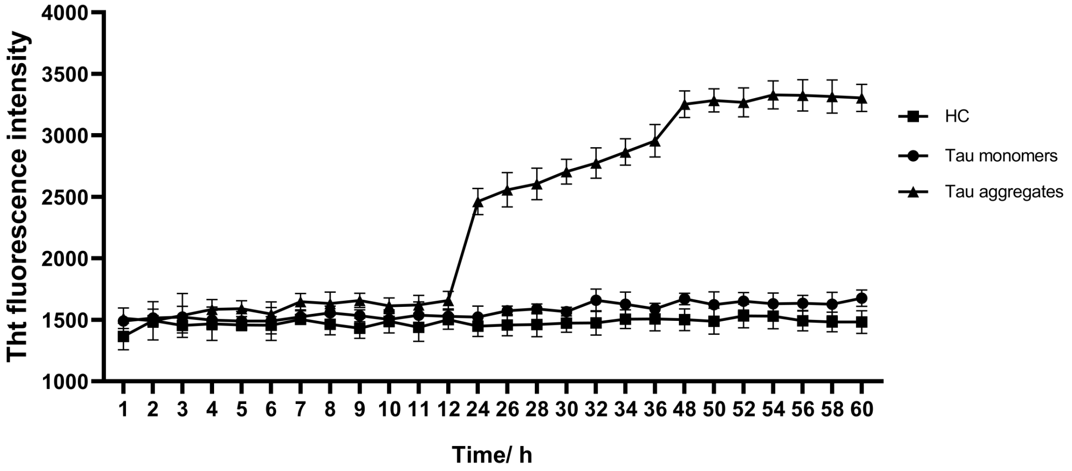

Figure S1. Tau monomers were induced to aggregate by heparin. ThT fluorescence intensity was measured at different time intervals. This experiment was repeated four times. The data are presented as mean ± SD.

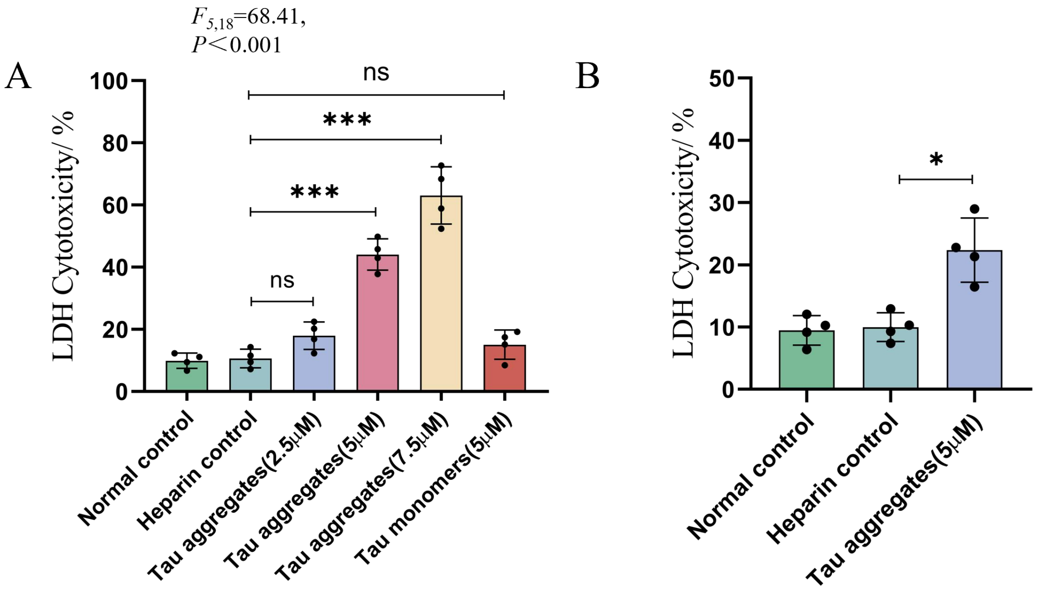

Figure S2. Measurement of the cytotoxicity of Tau aggregates to SH-SY5Y cells and HEK-293T cells. (A) The cytotoxicity of different concentrations of Tau aggregates to SH-SY5Y cells detected by LDH method. This experiment was repeated four times. (B) The cytotoxicity of Tau aggregates to the HEK-293T cells detected by LDH

method. This experiment was repeated four times. Data represent means ± SD and were analyzed by one-way ANOVA (A) or Student's t-test (B). *p<0.05, ***p<0.001, ns, not significant.

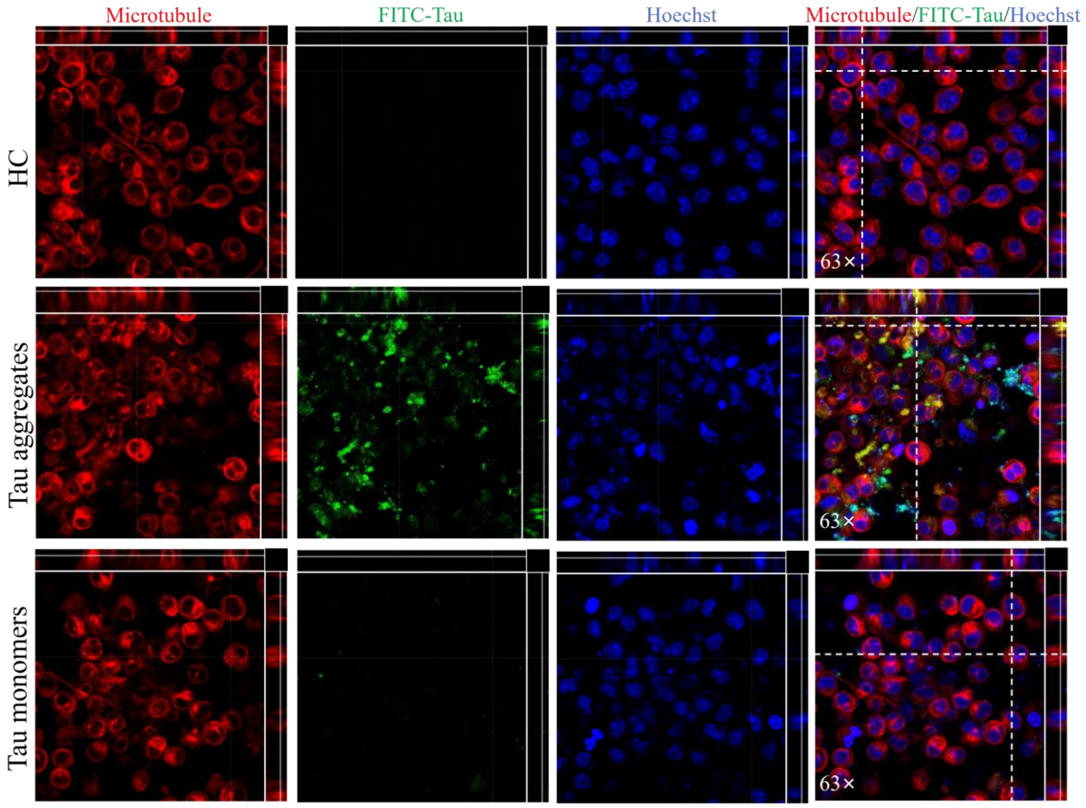

Figure S3. Extracellular Tau aggregates enter SH-SY5Y cells. Representative confocal images including multiple z-stacks showing colocalization of SH-SY5Y tubulin and FITC-Tau (Corresponding to Figure 2). Red fluorescence: microtubule stained with far-infrared fluorescence staining kit; Green fluorescence: FITC-Tau; Blue fluorescence: nuclei stained with Hoechst 33342. Scale bar, 10 μm.

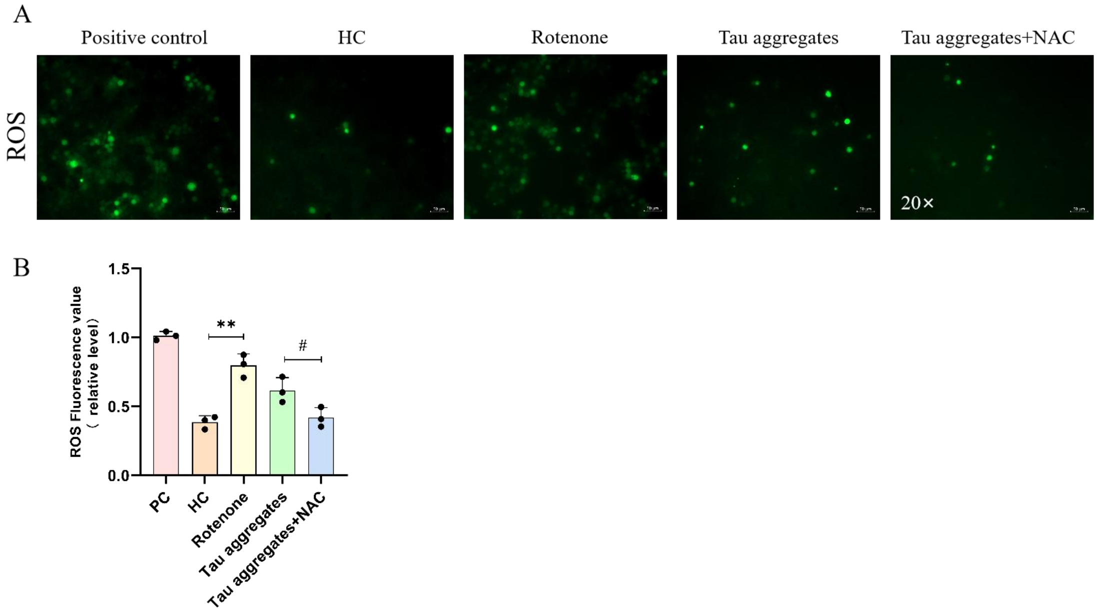

Figure S4. Rotenone activates ROS production, and NAC inhibits ROS production. (A) Representative fluorescence micrograph showing ROS levels. SH-SY5Y cells were treated with heparin (5 μM), rotenone (5 μM), Tau aggregates (5 μM) or Tau aggregates-NAC (Tau aggregates: 5 μM, NAC: 500 μM) for 24 h, after which the ROS levels were measured. (B) Bar chart of the relative ROS levels in Figure S4A. Scale bar, 50 μm. The experiment was performed in triplicate. Data represent means ± SD and were analyzed by Student's t-test. ***p*<0.01, #*p*<0.05.

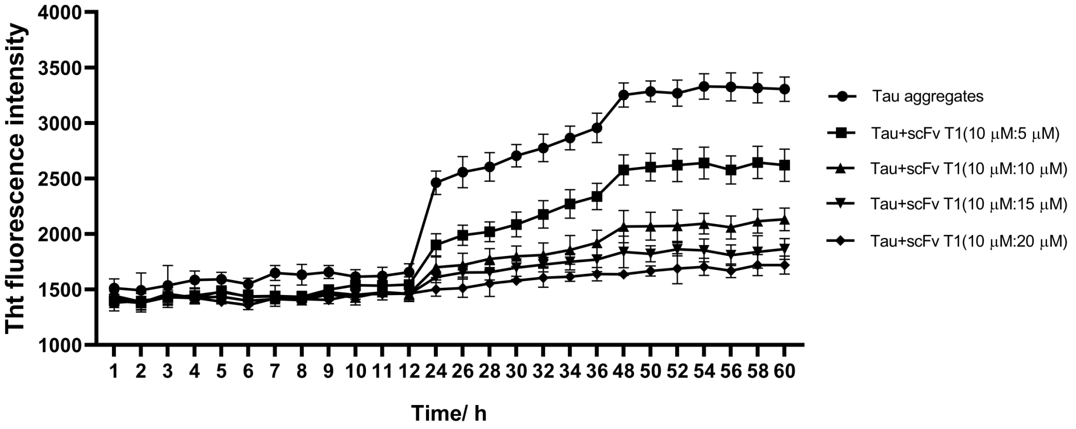

Figure S5. ScFv T1 inhibits extracellular Tau aggregation. Tau monomers were incubated with scFv T1 of different concentrations in the aggregation buffer. ThT

fluorescence intensity was measured at different time intervals. This experiment was repeated four times. The data are presented as mean ± SD.

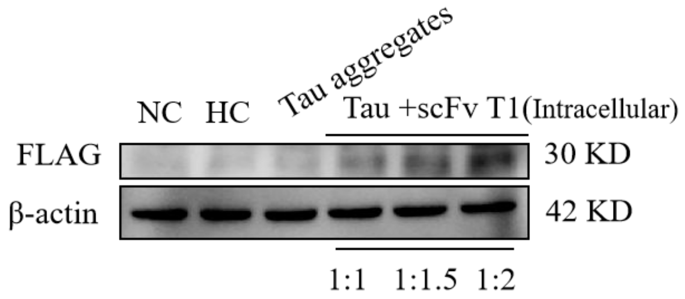

Figure S6. ScFv T1 was expressed in HEK-293T cells. The recombinant plasmids expressing Tau RD/P301S-ECFP, Tau RD/P301S-EYFP and scFv T1-FLAG were co-transfected into HEK-293T cells at different ratios (Tau RD/P301S-ECFP:Tau RD/P301S-EYFP:scFv T1= 1:1:0, 1:1:2, 1:1:3 and 1:1:4). The expression level of scFv T1-FLAG was measured by western blot. The grouping of blots cropped from different gels of same samples.

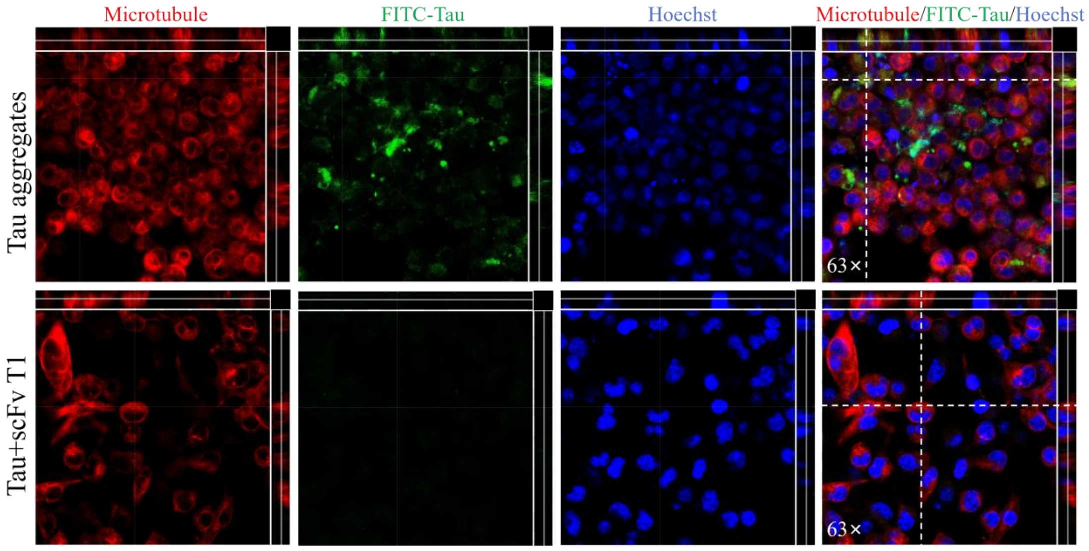

Figure S7. The number of Tau aggregates available for uptake by SH-SY5Y cells decreased after the inhibition of Tau aggregation by ScFv T1. Representative confocal images including multiple z-stacks showing colocalization of SH-SY5Y tubulin and FITC-Tau (Corresponding to Figure 8). Red fluorescence: microtubule stained with

far-infrared fluorescence staining kit; Green fluorescence: FITC-Tau; Blue fluorescence: nuclei stained with Hoechst 33342. Scale bar, 10 μm.
